# Challenges on solar oxidation as post-treatment of municipal wastewater from UASB systems: Treatment efficiency, disinfection and toxicity

**DOI:** 10.1016/j.scitotenv.2022.157940

**Published:** 2022-12-01

**Authors:** Fernando Rodrigues-Silva, Maria Clara V. M. Starling, Camila C. Amorim

**Affiliations:** Research Group on Environmental Applications of Advanced Oxidation Processes, Department of Sanitary and Environmental Engineering, Federal University of Minas Gerais, Av. Antônio Carlos, 6627, Belo Horizonte, Minas Gerais 31270-901, Brazil

**Keywords:** AOP, Advanced Oxidation Processes, ARB, Antibiotic-Resistant Bacteria, ARG, Antibiotic-Resistant Genes, CAS, Conventional Activated Sludge, CECs, Contaminants of Emerging Concern, CPC, Compound Parabolic Collector, DOM, Dissolved Organic Matter, ERA, Ecotoxicological Risk Assessment, HRT, Hydraulic Residence Time, LA, Latin America, MEC, Measured Environmental Concentration, MSE, Municipal Secondary Effluent, MWWTP, Municipal Wastewater Treatment Plant, NOEC, Non-Effect Concentration, NOM, Natural Organic Matter, PNEC, Predicted No Effect Concentration, QSAR, Quantitative Structure-Activity Relationship, ROS, Reactive Oxygen Species, RPR, Raceway Pond Reactor, RQ, Risk Quotient, SODIS, Solar Disinfection, TP, Transformation Product, UASB, Upflow Anaerobic Sludge Blanket, UASB + PT, UASB Followed by Post-treatment, WET, Whole Effluent Toxicity, AOP, Urban wastewater, Neutral pH, UASB post-treatment, Solar photo-Fenton

## Abstract

The application of solar photo-Fenton as post-treatment of municipal secondary effluents (MSE) in developing tropical countries is the main topic of this review. Alternative technologies such as stabilization ponds and upflow anaerobic sludge blanket (UASB) are vastly applied in these countries. However, data related to the application of solar photo-Fenton to improve the quality of effluents from UASB systems are scarce. This review gathered main achievements and limitations associated to the application of solar photo-Fenton at neutral pH and at pilot scale to analyze possible challenges associated to its application as post-treatment of MSE generated by alternative treatments. To this end, the literature review considered studies published in the last decade focusing on CECs removal, toxicity reduction and disinfection via solar photo-Fenton. Physicochemical characteristics of effluents originated after UASB systems alone and followed by a biological post-treatment show significant difference when compared with effluents from conventional activated sludge (CAS) systems. Results obtained for solar photo-Fenton as post-treatment of MSE in developed countries indicate that remaining organic matter and alkalinity present in UASB effluents may pose challenges to the performance of solar advanced oxidation processes (AOPs). This drawback could result in a more toxic effluent. The use of chelating agents such as Fe^3+^-EDDS to perform solar photo-Fenton at neutral pH was compared to the application of intermittent additions of Fe^2+^ and both of these strategies were reported as effective to remove CECs from MSE. The latter strategy may be of greater interest in developing countries due to costs associated to complexing agents. In addition, more studies are needed to confirm the efficiency of solar photo-Fenton on the disinfection of effluent from UASB systems to verify reuse possibilities. Finally, future research urges to evaluate the efficiency of solar photo-Fenton at natural pH for the treatment of effluents from UASB systems.

## Highlights

• UASB systems are frequently used in developing areas to treat municipal wastewater.
• Dissimilarity amidst UASB and CAS effluents challenges the use of solar AOPs.
• Lack studies on solar photo-Fenton application for UASB effluent improvement.
• Fractionated iron addition in photo-Fenton is promising for UASB effluent treatment.
• AOPs must be explored as UASB post-treatment strategies.

## Graphical abstract

**Figure:**
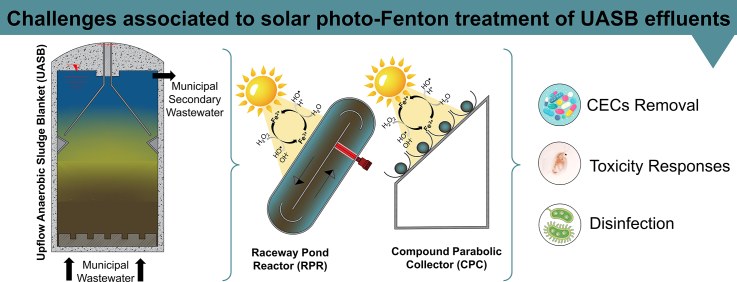

## 1 Introduction

Municipal wastewater treatment plants (MWWTP) worldwide aim to remove suspended particles, organic matter, nutrients and pathogens from domestic sewage (Giannakis et al., 2017) prior to discharge or reuse. However, most MWWTPs in developing countries lack a tertiary treatment stage due to restricted investments in sanitation. Thus, resulting in limited removal of nutrients and pathogens (Noyola et al., 2012). Moreover, in the absence of an advanced treatment stage, persistent compounds, especially non-biodegradable chemicals, may resist aerobic and anaerobic biological treatment processes, thus remaining in municipal secondary effluent (MSE).

Many recent studies have focused on the improvement of MSE quality by removing persistent compounds and pathogens and reducing toxicity to allow for its reuse in irrigation or a safer discharge to the environment (Costa et al., 2021b; Gonçalves et al., 2020; Maniakova et al., 2022; Rizzo et al., 2020). Most of these studies concern the removal of micropollutants (MPs) from MSE. MPs occur in low concentrations (ng L^−1^–μg L^−1^) in environmental matrices and constitute daily use products (licit and illicit drugs, hormones, personal care products and personal hygiene products, etc.) and their metabolites, all of which end up in municipal wastewater (Rizzo et al., 2019; Starling et al., 2019). Due to recent interest in the effects promoted by MPs on aquatic biota and human health and lack of legal standards related to their occurrence in the environment and removal from MSE, these pollutants have also been referred to as contaminants of emerging concern (CECs) (Arzate et al., 2020; Patel et al., 2019).

CECs may reach surface water via wastewater discharge generating potential risks for human health, animals, and the environment. Among CECs, pharmaceuticals have been associated to ecotoxicological risks (Sodré et al., 2018) and adverse effects upon aquatic organisms, depending on exposure route, bioavailability, susceptibility, and stability (Espinosa-Barrera et al., 2021). Marson et al. (2022) concluded that from the high load of CECs present in municipal wastewater and reaching surfaces waters, most are pharmaceutical drugs. In fact, concentrations of some pharmaceutical drugs (e.g. sulfamethoxazole and 17α-ethynylestradiol) were higher in the output of MWWTP when compared to the inlet due to difficulties associated to the analytical detection of low solubility compounds which are usually excreted in the conjugated form, yet present in detectable form in the outlet (Plósz et al., 2010; Queiroz et al., 2012). Hence, most studies regarding the application of solar photo-Fenton as post-treatment of MSE target the removal of pharmaceutical drugs.

**Fig. 1:**
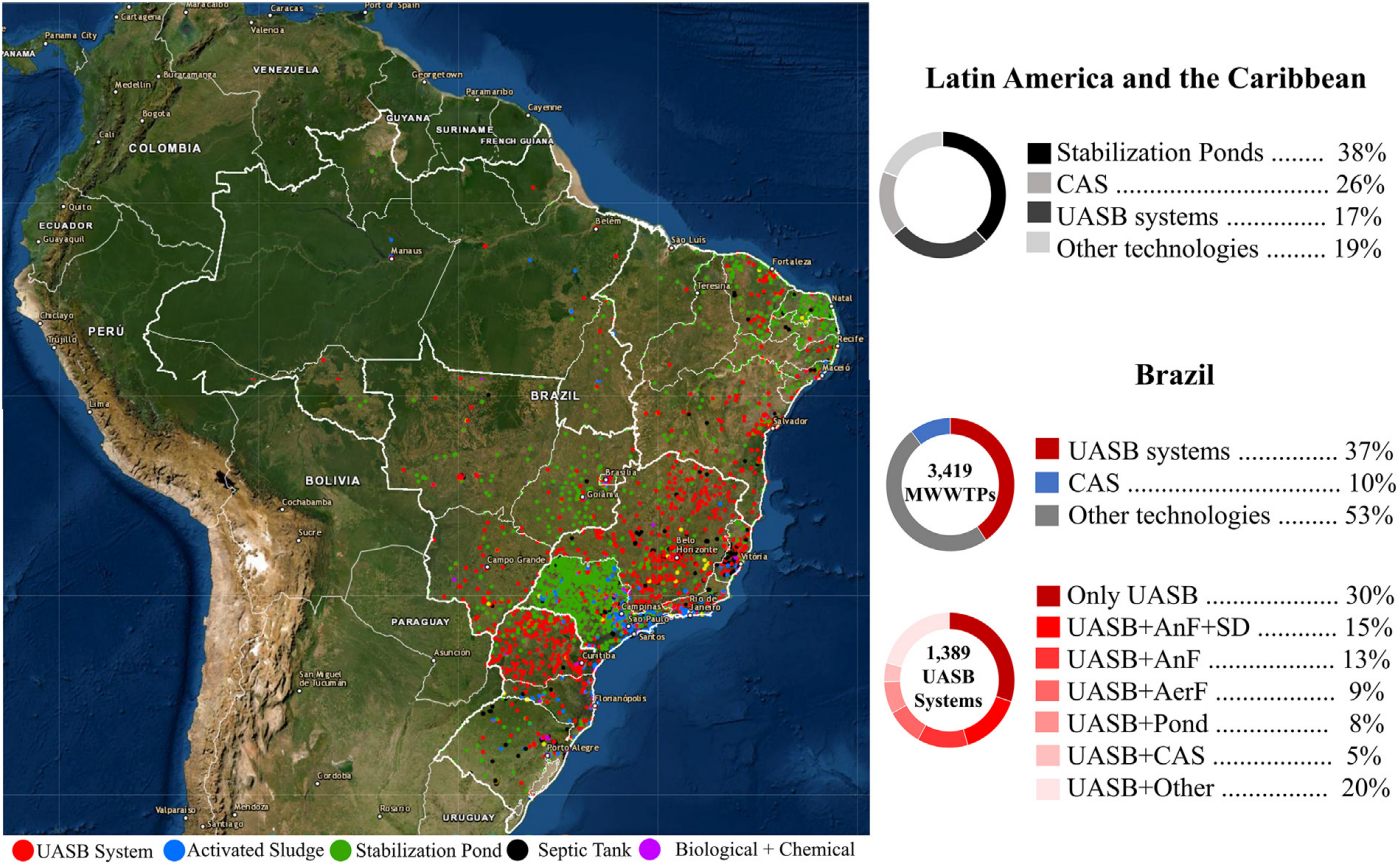
Wastewater Treatment Technologies applied in Brazil focused on Upflow Anaerobic Sludge Blanket reactors (UASB) followed by different systems. Note: UASB – Upflow anaerobic sludge blanket; CAS – conventional activated sludge; AnF – anaerobic filter; SD – secondary decanter; AeF – aerobic filter; pond – stabilization ponds.

Source: Adapted from ANA (2020).

Most of the published studies aiming at the application of solar photo-Fenton as post-treatment of MSE are performed with effluents from conventional activated sludge (CAS). However, results obtained in these studies do not apply to the reality in Latin America (LA), where main technologies applied in MWWTP are stabilization ponds (38 %), activated sludge (26 %), and Upflow Anaerobic Sludge Blanket (UASB) (17 %) (Noyola et al., 2012; Von Sperling, 2016). Fig. 1 illustrates the application of these technologies in LA with a special focus in the application of UASB reactors for the treatment of MWW in Brazil, as it is the biggest and most populous country in the region.

The number of UASB reactors applied for domestic wastewater treatment in Brazil (1389 units) is nearly four-fold higher than that of CAS reactors (359 units). UASB reactors correspond to 37 % of treatment units in Brazil, compared to 9 % for CAS (ANA, 2020). Considering other LAC countries, UASB reactors used for municipal wastewater treatment in Colombia, Dominican Republic, Guatemala and Mexico correspond to, respectively, 25 %, 18 %, 17 % and 10%of all existing MWWTP in these countries (Noyola et al., 2012). As MSE treated via anaerobic and aerobic processes are different as according to physicochemical quality (Oliveira and Von Sperling, 2008), it is critical to investigate the performance of advanced treatment technologies for the removal of CECs and pathogens for effluents generated from anaerobic treatment systems applied in LA.

Advanced oxidation processes (AOPs), such as UVC-H_2_O_2_, Fenton, photo-Fenton, ozonation, and UV-TiO_2_, are based on the generation of highly reactive oxidizing compounds such as hydroxyl radicals (HO•). These processes have been increasingly studied and implemented as post-treatment of MSE (Rizzo et al., 2019). Special attention has been given to solar irradiated AOPs (Malato et al., 2009) which represent valuable alternatives in developing countries located in tropical areas, such as Latin America, as these regions receive high levels of incident solar radiation and suffer from lack of investment in wastewater treatment facilities.

The solar photo-Fenton process is based on the generation of HO•. The reaction is catalyzed by iron in the presence of an oxidant (H_2_O_2_, persulfate) and cyclical oxidation/reduction of iron occurs in the system. The classic Fenton reaction (Eq. (1)) takes place in the dark, generating HO•. The process is enhanced under irradiation as the photolysis of ferric ions produces HO• and accelerates Fe^2+^ cycling (Eq. (2)). In addition, iron hydroxides present in the system absorb radiation in the visible range (<450 nm), thus corresponding to an additional route for the formation of HO• (Eq. (3)) (Pignatello et al., 2006).

(1) $${\mathrm{Fe}}^{2+}+H_{2}O_{2}\to {\mathrm{Fe}}^{3+}+{\mathrm{HO}}^{-}+\mathrm{HO}˙$$

(2) $${\mathrm{Fe}}^{3+}+H_{2}O+\mathrm{hv}\to {\mathrm{Fe}}^{2+}+\mathrm{HO}˙+H^{-}$$

(3) $$\mathrm{Fe}{\left(\mathrm{OH}\right)}^{2+}+\mathrm{hv}↔{\mathrm{Fe}}^{2+}+\mathrm{HO}˙$$

The photo-Fenton process is commonly conducted at acid pH as hydrated iron ions predominate in this pH (FeOH^2+^,48 %, pH 2.8 and 25 °C, 0.5 M of ionic strength), thus corresponding to maximum efficiency of the H_2_O_2_ catalysis reaction (Pignatello et al., 2006). Despite better performance under acidic conditions, pH adjustment prior and after treatment may increase process costs and risks associated with the storage of chemical reagents and sludge disposal. Hence, there is high interest in performing solar photo-Fenton at circumneutral pH and the scientific community has proposed different strategies for this purpose.

Despite broad use of anaerobic wastewater treatment systems in LA and the disparity between effluents generated from anaerobic and aerobic wastewater treatment processes, most studies have applied solar photo-Fenton as post-treatment of CAS effluent. Thereafter, there is a gap in the literature regarding the assessment of solar photo-Fenton performance towards CEC removal, disinfection, and toxicity reduction in effluents from anaerobic treatment systems. Considering intense application of anaerobic treatment systems in tropical developing countries and challenges posed by natural organic matter and ions present in these effluents upon AOPs (Lado Ribeiro et al., 2019), it is critical to elucidate the effectiveness of solar photo-Fenton as post-treatment of anaerobic effluents.

In this context, this review aims (i) to compare the quality of effluents resulting from domestic wastewater treatment via CAS systems operating worldwide with UASB systems operating mainly in LAC; and (ii) to discuss challenges associated to post-treatment of UASB effluents via solar photo-Fenton in light of studies carried out worldwide to assess the removal of pharmaceutical drugs and pathogens from MSE, as well as toxicity responses. This content is valuable for evaluating the feasibility of solar photo-Fenton application as post-treatment of MSE from anaerobic treatment systems.

## 2 Quality of MSE originated from CAS and anaerobic systems

**Fig. 2:**
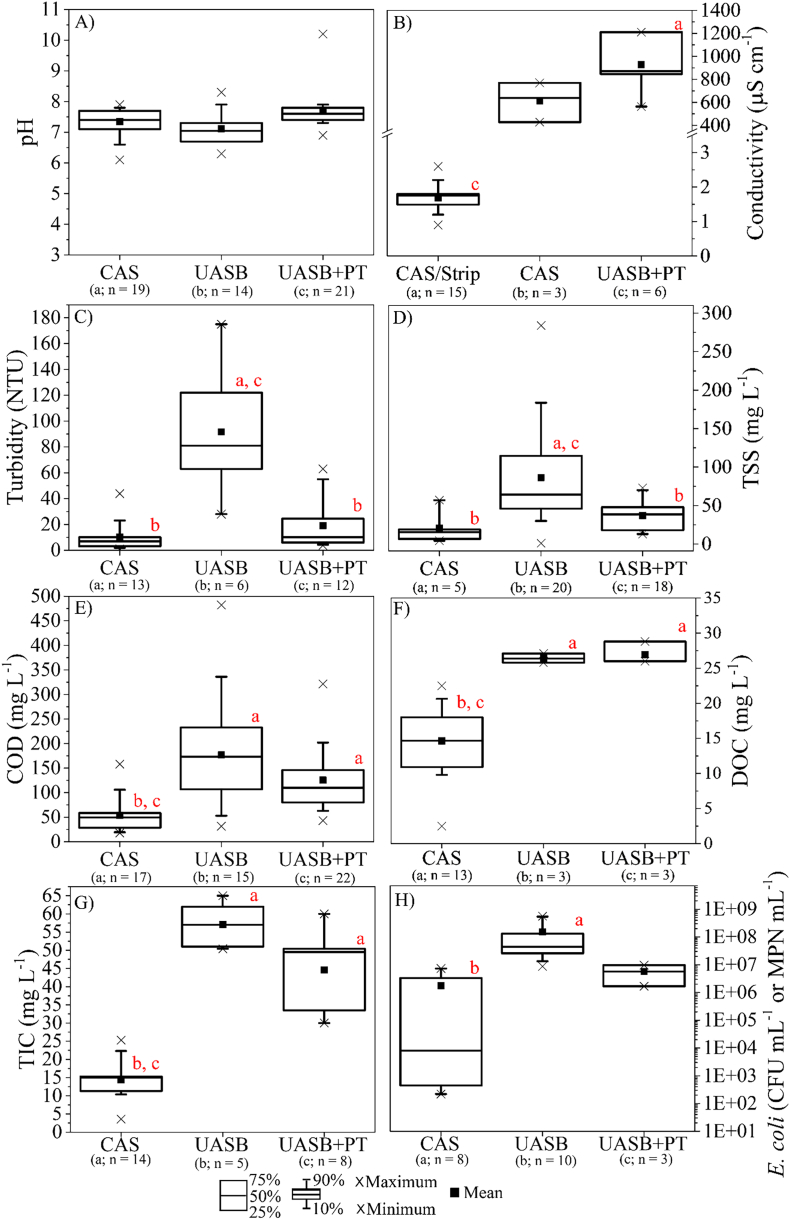
Physicochemical and biological characteristics of effluents generated from the treatment of municipal wastewater by conventional activated sludge (CAS), upflow anaerobic sludge blanket reactor (UASB), upflow anaerobic sludge blanket followed by post-treatment (UASB + PT): A) pH; B) conductivity; C) turbidity; D) total suspended solids (TSS); E) chemical oxygen demand (COD); F) dissolved organic carbon (DOC); G) total inorganic carbon (TIC); H) *Escherichia coli*. The letters a, b, and c indicate the significant difference (α = 0.05). Note: CAS/Strip shows the prior acidification with H_2_SO_4_ to perform (HCO_3_^−^/CO_3_^−2^) strip regarding the solar photo-Fenton post-treatment.

Physicochemical quality of effluents originated from secondary municipal wastewater treatment plants which apply CAS, UASB and UASB followed by post-treatment (UASB + PT) systems was investigated by a literature review using the keywords “UASB effluent” AND “post-treatment UASB effluent” AND “quality”. All data regarding UASB effluent quality presented in Fig. 2 were obtained from UASB systems operating in developing countries (Aiyuk et al., 2004; Amildon Ricardo et al., 2018; Antonio da Silva et al., 2021; Bhatti et al., 2014; Bressani-Ribeiro et al., 2021a; Cardinali-Rezende et al., 2013; De Almeida et al., 2009; Espinosa et al., 2021; Gonçalves et al., 2020; Komolafe et al., 2021; Lopes et al., 2017; Luo et al., 2014; Marson et al., 2020; Oliveira and Von Sperling, 2008; Paniagua et al., 2020; Silva et al., 2021a; Vassalle et al., 2020a, Vassalle et al., 2020). According to these studies, post-treatment systems following UASB reactors include aerated filter, anaerobic filter, trickling filter, facultative pond, maturation pond and, to a lesser extent, coagulation/flotation unit. No data was available for UASB effluent quality in developed countries as the application of UASB in these countries is limited to the treatment of industrial wastewater (Chan et al., 2009). Yet, physicochemical quality data for MSE derived from CAS systems operated in developed regions is abundant. Data on CAS effluent quality (Fig. 2) was extracted from studies related to CAS systems operating worldwide (Arzate et al., 2020, Arzate et al., 2017; Costa et al., 2020; De la Obra Jiménez et al., 2019; Dimane and El Hammoudani, 2021; Freitas et al., 2017; Salaudeen et al., 2018; Soriano-Molina et al., 2021, Soriano-Molina et al., 2019b, Soriano-Molina et al., 2019a; Starling et al., 2021a; Vilela et al., 2022).

Statistical analysis was performed using the BioEstat 5.3 free software (Tefé, Amapá, Brazil). Physicochemical and biological characteristics of effluents from CAS, UASB and UASB + PT were compared by Kruskal-Wallis (5 % significance level, α = 0.05) followed by the Dunn non-parametric multiple comparisons test (5 % significance level, α = 0.05). Regarding dissolved organic carbon (DOC) and total inorganic carbon (TIC), significant differences were assessed by using Mann-Whitney (5 % significance level, α = 0.05) non-parametric test as there was no data available for the UASB effluent.

### 2.1 Effect of anaerobic effluents constituents upon solar photo-Fenton: operational conditions

#### 2.1.1 pH

No significant difference was detected between the pH of MSE from aerobic and anaerobic treatment systems (Fig. 2A). As all secondary treatment systems generate effluents which present circumneutral pH, efforts to perform solar photo-Fenton as a post-treatment at neutral pH by using the intermittent iron addition strategy (Carra et al., 2014; Freitas et al., 2017; Starling et al., 2021a) or complexing agents (Arzate et al., 2020; Costa et al., 2020, Costa et al., 2021a; Maniakova et al., 2020; Silva et al., 2021a; Soriano-Molina et al., 2021, Soriano-Molina et al., 2019a, Soriano-Molina et al., 2019b, Soriano-Molina et al., 2018) must be encouraged to increase treatment applicability.

Carra et al. (2013) proposed the sequential iron addition strategy to allow for availability of dissolved iron throughout the entire reaction. Freitas et al. (2017) and Starling et al. (2021a) reported effective degradation of CECs from secondary wastewater by solar photo-Fenton at neutral or circumneutral pH using single and sequential iron additions, respectively. Alternatively, the use of iron complexing agents, which increase iron solubility to a broader pH range, also allows the performance of the photo-Fenton process at neutral pH. Poly-carboxylate acids such as oxalic acid, citric acid, ethylenediamine-*N*,*N*′-disuccinic acid (EDDS), ethylene-diamine-tetra-acetic acid (EDTA), and nitrilotriacetic acid (NTA) are commonly applied for this purpose (Arzate et al., 2020; Soriano-Molina et al., 2019b).

Ahile et al. (2020a) applied the iron chelation strategy for the treatment of effluents in the photo-Fenton process at circumneutral pH. Among the positive aspects of using this strategy in the photo-Fenton process are (i) H_2_O_2_ activation with the generation of HO• still takes place in the system; (ii) enhanced iron solubility at circumneutral pH promoting faster reduction of Fe^3+^ to Fe^2+^ under irradiation; (iii) reactions between complexes and HO^0^ form species which present higher quantum yield when compared to those formed in the presence of Fe^3+^ (Dias et al., 2014; Moreira et al., 2017). Eqs. (4), (5) demonstrate the generalized interaction between light and chelating agents (Fe^3+^-L). Eq. (4) represents the photo-activation of the Fe^3+^-L complex which promotes a ligand to metal charge transfer, thus reducing Fe^3+^ to Fe^2+^ and allowing for a more profitable catalytic cycle of iron. In addition to this mechanism, organic radicals (L•) produced in the system may generate other reactive oxygen species (ROS) as shown in Eq. (5) (Papoutsakis et al., 2015a, Papoutsakis et al., 2015b).

(4) $${\mathrm{Fe}}^{3+}-L_{n}+\mathrm{hv}\to {\mathrm{Fe}}^{2+}-L_{n-1}+L˙$$

(5) $$L˙+O_{2}\to L^{+}+O_{2}{˙}^{-}\to \to \to H_{2}O_{2}$$

Studies demonstrate a great interest in the use of Fe^3+^-EDDS to promote CECs removal from MSE via solar photo-Fenton (Maniakova et al., 2022, Maniakova et al., 2021; Miralles-Cuevas et al., 2021; Sánchez-Montes et al., 2022; Sánchez Pérez et al., 2020). According to Prada-Vásquez et al. (2021), this is the most popular version of solar photo-Fenton process at neutral pH for post-treatment of MSE. Mechanisms of EDDS action as a chelating agent in photo-Fenton process are shown in Eqs. (6), (7), (8), (9), (10), (11) and are related mainly to simultaneous effect of EDDS•^3−^, H_2_O_2_, HO• and O_2_•^−^ (Soriano-Molina et al., 2019a, Soriano-Molina et al., 2018; Wu et al., 2014). The use and efficiency of Fe^3+^-EDDS in solar photo-Fenton for post-treatment of MSE is deeply discussed in Section 3.

(6) $${\mathrm{Fe}}^{3+}-\mathrm{EDDS}\;\overset{\mathrm{HV}}{\to }\;{\mathrm{Fe}}^{2+}+\mathrm{EDDS}$$

(7) $${\mathrm{Fe}}^{3+}-\mathrm{EDDS}+H_{2}O_{2}\overset{\mathrm{hv}}{\to }{\mathrm{Fe}}^{2+}+\mathrm{EDDS}˙+\mathrm{HO}{˙}_{2}/O{˙}_{2}^{-}+H^{+}$$

(8) $$\mathrm{EDDS}˙+O_{2}\to {\mathrm{EDDS}}_{\mathrm{ox}}+H_{2}O_{2}$$

(9) $$O{˙}_{2}^{-}+H^{+}↔\mathrm{HO}{˙}_{2}$$

(10) $$O{˙}_{2}^{-}+\mathrm{HO}{˙}_{2}\to H_{2}O_{2}+O_{2}$$

(11) $$\mathrm{HO}{˙}_{2}+\mathrm{HO}{˙}_{2}\to H_{2}O_{2}$$

Despite advantages associated to photo-Fenton operation at neutral pH, some studies suggest prior effluent acidification with H_2_SO_4_ for the consumption of carbonates and bicarbonates from CAS effluents. Total inorganic carbon (TIC) concentrations below 15 mg L^−1^ are desirable to achieved enhanced efficiency during AOP treatment (Arzate et al., 2020, Arzate et al., 2017; Costa et al., 2020, Costa et al., 2021a; De la Obra Jiménez et al., 2019; Freitas et al., 2017; Soriano-Molina et al., 2021, Soriano-Molina et al., 2019a, Soriano-Molina et al., 2019b). These authors highlight that prior acidification strips carbonates and bicarbonates from MSE, which is advantageous as these molecules act as oxidative radical scavengers by (i) reacting with free radicals to generate secondary radicals with lower oxidative-reduction potential or (ii) competing for free radicals with target CECs (Lado Ribeiro et al., 2019).

#### 2.1.2 Alkalinity and conductivity

Effluents from UASB and UASB + PT (post-treatment) show significantly higher TIC (57 and 44 mg L^−1^; *p*-value 0.0002 and 0.0032, respectively) when compared to CAS effluent (15 mg L^−1^) (Fig. 2G). In contrast, no significant difference was detected between UASB and UASB + PT (p-value > 0.05). Monitoring of UASB and UASB + PT (trickling filter) for over 300 days (2019–2020) resulted in mean bicarbonate alkalinity values equivalent to 237(38) mg L^−1^ and 19(15) mg L^−1^ for UASB and UASB + PT effluents, respectively (Bressani-Ribeiro et al., 2021b). Conductivity is also higher (p-value < 0.05) for UASB + PT when compared to CAS + Strip (Fig. 2B).

As bicarbonates and other ions act as HO• scavengers and reduce AOP effectiveness (Lado Ribeiro et al., 2019), this may be an important limiting factor to the application of solar photo-Fenton as post-treatment of UASB and UASB + TP effluents. Rommozzi et al. (2020) evaluated the effect of different inorganic ions and their concentration upon disinfection via solar photo-Fenton process at near-neutral pH (1 mg Fe^2+^ L^−1^ and 10 mg H_2_O_2_ L^−1^). The authors reported a more significant inhibiting effect for HCO_3_^−^ ions, which presented adverse effects at concentrations ranging from 10 to 100 mg L^−1^. For Cl^−^, concentrations higher than 100 mg L^−1^ caused inhibitory effects upon treatment. Although this work evaluated disinfection of natural water matrices rather than MSE, it stands out for demonstrating the interference of organic matter and inorganic ions, especially HCO_3_^−^, on the oxidative efficiency of the photo-Fenton process. Despite scarcity of data regarding inorganic carbon concentration in UASB effluents, conductivity values, TIC and HCO_3_^−^ concentrations shown in Fig. 2B indicate that acidification may be required to reduce bicarbonates concentration prior the application of photo-Fenton process aiming at CEC removal from UASB effluent as this was proven to be an effective strategy for post-treatment CAS effluents (Klamerth et al., 2010).

#### 2.1.3 Alternative oxidants

Another strategy to reduce the scavenging effect of ions present in MSE, such as carbonate and bicarbonate, is the application of alternative radicals, which still have high oxidative potential yet are more selective than hydroxyl radicals. This is the case of sulfate radical (SO_4_^−^•), which is generated during solar photo-Fenton when hydrogen peroxide is replaced by either persulfate (S_2_O_8_^2−^) or peroxymonosulfate (HSO_5_^−^) as according to Eqs. (12), (13), (14), (15) (Ferreira et al., 2020; Starling et al., 2021b). As reaction rates between SO_4_^−^• and matrix components are lower than those observed for HO•, this may be a feasible strategy to enable solar photo-Fenton treatment of MSE from anaerobic treatment systems.

(12) $${S_{2}O_{8}}^{2-}+{\mathrm{Fe}}^{2+}\to {\mathrm{Fe}}^{3+}+2{{\mathrm{SO}}_{4}}^{-}˙$$

(13) $${S_{2}O_{8}}^{2-}+\mathrm{hv}\to 2{{\mathrm{SO}}_{4}}^{-}˙$$

(14) $${\mathrm{HSO}}_{5^{-}}+{\mathrm{Fe}}^{2+}\to {\mathrm{Fe}}^{3+}+{{\mathrm{SO}}_{4}}^{-}˙+{\mathrm{OH}}^{-}$$

(15) $${\mathrm{HSO}}_{5^{-}}+{\mathrm{Fe}}^{3+}\to {\mathrm{Fe}}^{2+}+{{\mathrm{SO}}_{5}}^{-}˙+H^{+}$$

In addition, HSO_5_^−^ (E^0^ = 1.81 V/SCE) is used as a strong oxidant to degrade organic compounds as it is easily activated by solid catalysts, heat, UV irradiation, among others, to generate SO_4_^−^• (Eq. (16)). HSO_5_^−^ redox potential (E^0^ = 2.44 V/SCE, pH 0) increases to 3.1 V/SCE in alkaline matrices, where it can be hydrolyzed to HO•. Therefore, co-occurrence of HO• and SO_4_^−^• may happen depending on matrix pH.

(16) $${{\mathrm{SO}}_{4}}^{-}˙+H_{2}O_{2}\to {{\mathrm{SO}}_{4}}^{2-}+\mathrm{HO}˙+H^{+}$$

Khan et al. (2021) reported that inorganic ions (CO_3_^2−^, HCO_3_^−^, Cl^−^ and SO_4_^2−^) affected the removal of target CECs in the presence of sulfate radicals. This effect was mainly associated to HCO_3_^−^ and CO_3_^2−^ which exert significant SO_4_•^−^ quenching through reactions shown in Eqs. (17), (18).

(17) $${{\mathrm{SO}}_{4}}^{-}˙+{{\mathrm{CO}}_{3}}^{2-}\to {{\mathrm{CO}}_{3}}^{-}˙+{{\mathrm{SO}}_{4}}^{2-}$$

(18) $${{\mathrm{SO}}_{4}}^{-}˙+H{{\mathrm{CO}}_{3}}^{-}\to {{\mathrm{CO}}_{3}}^{-}˙+{{\mathrm{SO}}_{4}}^{2-}+H^{+}$$

The use of persulfate as an oxidant in solar photo-Fenton at natural pH (intermittent iron additions) without prior removal of bicarbonates was successful for the removal of CECs (60 % removal), disinfection (reduction of 3 log units of *E. coli*), and elimination of antibiotic-resistant bacteria (2–4 log units) from CAS effluent (Starling et al., 2021a). Median conductivity of MSE was 638 μS cm^−1^ which is similar to that observed for UASB + PT effluents (871.5 μS cm^−1^). Thus, suggesting that this strategy may be effective for the improvement of MSE from UASB systems. Despite lack of data related to the conductivity of UASB effluent, mean conductivity of UASB effluent was equivalent to 841 μS cm^−1^ (Bhatti et al., 2014), which is similar to values observed for UASB + PT. In contrast to observations made for solar photo-Fenton in the presence of hydrogen peroxide, these results indicate that solar photo-Fenton treatment using persulfate is a promising strategy for post-treatment of UASB effluent with no need for prior acidification.

#### 2.1.4 Turbidity and suspended solids

Turbidity and total suspended solids (TSS) may limit the application of photocatalytic processes due to the light scattering effect (Ma et al., 2021). Consequently, treatment times are increased compared to the time required to achieve the same efficiency in clear effluents (Costa et al., 2021b). There is no significant difference between turbidity of CAS effluents (6.9 NTU; *n* = 13) and UASB + PT effluents (10.1 NTU; *n* = 12) (α = 0.05) (Fig. 2C). However, UASB effluents (*n* = 6) showed median turbidity equivalent to 81 NTU resulting in significant difference when compared to CAS (*p*-value 0.0005) and UASB + PT effluents (p-value 0.0242).

Similarly, no significant difference (p-value 0.6679) was detected for TSS concentration in CAS effluents (15.5 mg L^−1^) compared to UASB + PT effluents (38.5 mg L^−1^). Yet, TSS concentration in the output of UASB effluents (64.2 mg L^−1^) is significantly higher than TSS concentrations in CAS effluent (p-value 0.0061) and UASB + PT effluents (p-value 0.0113). These results indicate that direct post-treatment of UASB effluent via irradiated processes will probably require a previous stage to reduce suspended solids concentrations to allow for process improvement and reduction of treatment time.

#### 2.1.5 Natural organic matter content

Natural organic matter (NOM) present in MSE may consume oxidative radicals generated during AOP, thus decreasing the removal of target contaminants (Lado Ribeiro et al., 2019). Quantification of organic matter in effluents may be measured (i) directly by using a Total Organic Carbon (TOC) Analyzer, (ii) or indirectly by quantification of oxygen required for biological (Biological Oxygen Demand, BOD) or chemical oxidation (Chemical Oxygen Demand, COD) of organic matter. Direct quantification is not affected by NOM biodegradability nor by the presence of inorganic ions and other components. Nevertheless, most studies related to the characterization of UASB effluents quantify organic content by measuring COD, probably due to lack of access to a TOC Analyzer (Fig. 2E) and absence of legal standards associated to Total Organic Carbon or Dissolved Organic Carbon (DOC) for wastewater disposal in developing countries. Effluents from CAS systems showed dissolved organic carbon (DOC) levels below 23 mg L^−1^ (median = 14.7 mg L^−1^), while effluents from UASB and UASB + PT showed average concentrations equivalent to 26.5 and 30.3 mg L^−1^, respectively. According to the few reports of DOC concentrations in UASB effluents, organic content in MSE from UASB systems differs from MSE from CAS systems (*p*-value 0.0288 for UASB and 0.0219 for UASB + PT). Considering COD values, CAS effluents average 49.5 mg L^−1^, compared with 173 mg L^−1^ for UASB effluents, and 110 mg L^−1^ in UASB + PT effluents. COD concentrations were significantly lower in the output of CAS compared to UASB (p-value 0.0003) and UASB + PT (p-value 0.0009). According to Costa et al. (2021b), NOM present in the matrix affects the degradation of CECs and disinfection of wastewater via photo-Fenton (Eq. (19)) because NOM can act as scavengers of HO• or compete with the target CECs.

(19) $$\mathrm{NOM}+\mathrm{HO}˙\to {\mathrm{NOM}}_{\mathrm{OX}}+{\mathrm{HO}}_{2}/O_{2}{˙}^{-}$$

Therefore, optimum reagent concentrations required for the removal of CEC from UASB and UASB + PT effluents via solar photo-Fenton are probably higher than those applied for post-treatment of CAS effluents.

Among LA, Brazil is the major user of UASB reactors for municipal wastewater treatment. 77 % of all MWWTP in operation in Brazil consist of stabilization ponds, followed by UASB reactors (Chernicharo et al., 2018). However, according to the bibliometric survey performed by Costa et al. (2021b), only very few studies have investigated the use of solar photo-Fenton as post-treatment of MSE generated by anaerobic treatments. Therefore, data regarding solar photo-Fenton performed with UASB and UASB + TP effluents is scarce. Organic matter removal achieved by UASB is limited to 65–75 %, and the removal of pathogens ranges between 90 % and 99 %. Considering typical initial concentration of pathogen indicators in raw municipal wastewater (1 × 10^9^ CFU mL^−1^), final concentrations equivalent to 1 × 10^8^ (1 Log removal) or 1 × 10^7^ CFU mL^−1^ (2 Logs removal), respectively, are higher than concentrations set for environmentally safe discharge. Therefore, a removal rate equivalent to 99 % is still not satisfactory as it results in a final concentration equivalent to 1 × 10^7^–10^4^ CFU mL^−1^ (Chernicharo, 2015; Sperling, 2008).

Median *E. coli* concentrations were significantly different (*p*-value 0.003) in CAS effluents (2.3 × 10^4^ CFU mL^−1^ or MPN 100 mL^−1^) compared to UASB effluents (6.5 × 10^7^ CFU mL^−1^ or MPN 100 mL^−1^). No significant difference occurs between *E. coli* concentrations in effluents from UASB and UASB + PT (4.0 × 10^6^ CFU mL^−1^ or MPN 100 mL^−1^) (Fig. 2H). These results suggest that disinfection of UASB effluents via solar photo-Fenton will be more challenging when compared to CAS effluents. Considering broad applicability of UASB systems in developing countries and proved efficiency of solar photo-Fenton for disinfection of MSE, as well as the urgent need to remove antibiotic-resistant bacteria (ARB) and genes (ARG) from MSE, it is urgent to evaluate the efficiency of solar AOPs for disinfection and removal of antimicrobial resistance from UASB effluents (Starling et al., 2021b). In this sense, photo-Fenton process performed as post treatment of MSE may improve the quality of effluents providing safer discharge to the environment and decreasing risks of waterborne diseases, thus improving public health.

#### 2.1.6 Nutrient content

UASB reactors are not effective to remove nutrients, presenting negative removal of nitrogen (−13 %) and phosphorus (−1 %) (Oliveira and Von Sperling, 2008). As these are inorganic ions, their occurrence in MSE limits the performance of photo-Fenton due to secondary consumption of hydroxyl radicals (Lado Ribeiro et al., 2019). According to Giannakis et al. (2021), phosphates are unreactive compounds that act as terminal receptors and do not propagate further reactions with HO•. Among 333 MWWTP comprised of UASB reactors in Brazil, only 25 % (82 plants) contain trickling filters (TF) as a post-treatment unit (Chernicharo et al., 2018). Although there is no available substantial data on the removal of nitrogen via UASB + PT, phosphorus removal is improved by 23 % when UASB is coupled to an aerated filter, anaerobic filter, trickling filter, flotation unit, facultative pond or maturation ponds (Oliveira and Von Sperling, 2008). Therefore, the efficiency of solar photo-Fenton must be evaluated not only for UASB effluents yet also for each of these multistage systems to cover the existing knowledge gaps.

Bressani-Ribeiro et al. (2018) reviewed the main characteristics and performance of rock and plastic-bed trickling filters following UASB reactors at laboratory, pilot, and full-scale for the treatment of MWW. Biochemical oxygen demand (BOD_5_) values ranged from 40 to 140 mg L^−1^ (median = 96 mg L^−1^), TSS concentration varied broadly between 35 and 181 mg L^−1^ (median = 49 mg L^−1^), and NH_4_^+^ concentrations were between 19 and 37 mg L^−1^ (median = 30 mg L^−1^). Median values presented for these parameters after post-treatment by TF (32 mg L^−1^ of BOD_5_, 22 mg L^−1^ of TSS, 19 mg L^−1^ of NH_4_^+^) reveal effluent quality improvement. This improvement may pave the way for the application of solar photo-Fenton as post-treatment of UASB + TF effluents.

Despite removing nitrogenous compounds, solar photo-Fenton applied as post-treatment for UASB-TF enable (i) oxidation of nitrogen compounds to NO_3_^−^, and (ii) photoreduction of NO_3_^−^ to NO_2_^−^ with the formation of NO_2_• (Zuo and Deng, 1998). Nitrate absorbs UVC radiation more readily than H_2_O_2_ (wavelengths < 250 nm), while nitrite absorbs mainly in the UVA region and extends into the visible region (Vinge et al., 2020). However, the generation of NO_2_• is not advantageous in Fenton-based process as it presents lower redox potential compared to HO• (Lado Ribeiro et al., 2019). Moreover, there still are uncertainties regarding inhibition of photo-Fenton process by natural components of wastewater. The nitration pathway involving the HO• + NO_2_^−^ mechanism could also induce the formation of aromatic nitrocompounds involving the addition of NO_2_• (Carlos et al., 2010).

Among all physicochemical parameters presented for CAS, UASB and UASB + PT effluents, NOM plays a critical role in the application of the photo-Fenton processes as post-treatment of MSE. Data gathered here indicate the need for a biological post-treatment following UASB reactors due to limited organic matter (COD and BOD_5_) removal achieved by UASB alone. In addition, it is urgent to evaluate strategies to allow for the application of photo-Fenton process as post-treatment of UASB and UASB + PT effluents, as matrix components may hinder the removal of CECs and disinfection and influence toxicity responses.

## 3 Predicting challenges to be faced during post-treatment of anaerobic effluents via solar photo-Fenton

There has been a rise in the interest towards the application of homogeneous solar photocatalysis to remove CECs present in aqueous matrices for the last decade. However, full-scale application of photo-Fenton as post-treatment of MSE is not yet a reality due to various reasons: (i) the numerous chemical compounds in fluctuating concentrations in MSE which hinders the surveillance of the totality of CECs; (ii) lack of enforcement in wastewater treatment facilities due to absence of explicit regulations aiming at the control and elimination of CECs from MSE; (iii) the presence of organic and inorganic scavengers as natural components of MSE (NOM, ions, etc.); (iv) and lack of accessible information associated to the design of photoreactors (Klavarioti et al., 2009; Maniakova et al., 2020; Schwarzenbach et al., 2006).

As CECs occur in fairly low concentrations in the environment, limits of detection (LOD) and quantification (LOQ) make the analysis of CEC complex and costly. Despite these limitations, plenty of effort has been put into research projects conducted at pilot scale to simulate real circumstances imposed by post-treatment of MSE (Wang et al., 2020). Only a few studies carried out in developed countries, assess the performance of photo-Fenton considering the occurrence and removal of CECs in their actual concentrations in MSE (Arzate et al., 2020, Arzate et al., 2017; Freitas et al., 2017; Soriano-Molina et al., 2021, Soriano-Molina et al., 2019b, Soriano-Molina et al., 2019a). In addition, while most studies are performed in solar chambers which simulate sunlight irradiation, recent studies have used solar photo-reactors operating at batch and continuous modes.

### 3.1 Solar photoreactors and operation settings

**Fig. 3:**
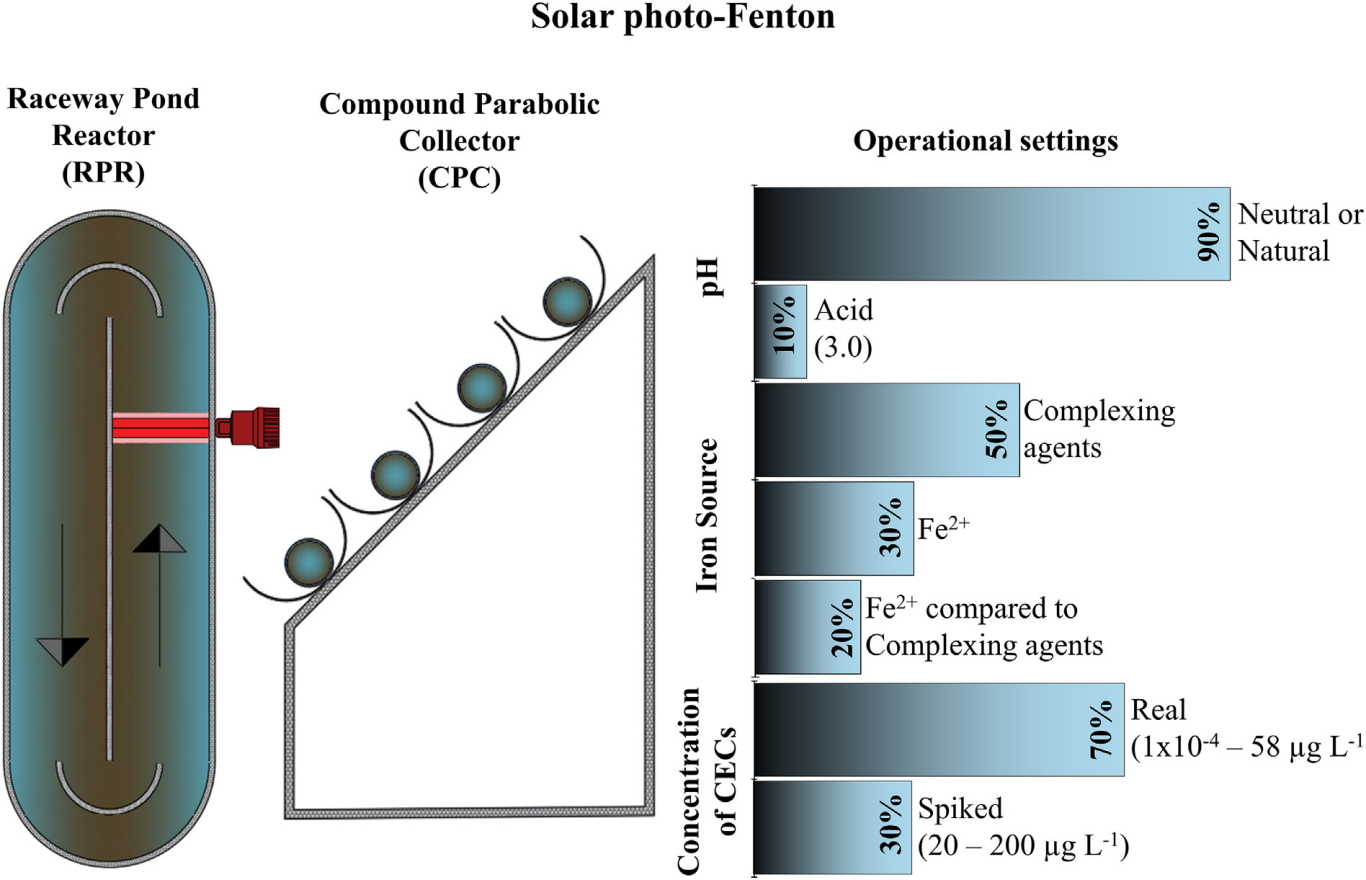
Schematic representation of the Raceway Pond and Compound Parabolic Reactors and operational settings used in studies evaluated in this review and related to the application of solar photo-Fenton at pilot-scale for the treatment of municipal wastewater aiming at CEC removal.

Solar photo Fenton is an alternative to remove CECs occurring in MSE. Among photoreactors designed to perform wastewater treatment via solar photocatalytic processes, options include reflective concentrating parabolic surfaces and non-concentrating collectors (Cabrera-Reina et al., 2019; Malato et al., 2007). Compound parabolic collectors (CPCs) are the most extensively low-concentrating models applied for the application of solar photo-Fenton. They consist of two connected parabolic reflective surfaces and an absorber tube in the axis. Besides the CPC, published studies have reported the use of a non-concentrating low-cost raceway pond reactor (RPR) as a sound system for the performance of solar photo-Fenton aiming at the removal of CECs from wastewater (Arzate et al., 2017; Costa et al., 2020, Costa et al., 2021a; Freitas et al., 2017; Soriano-Molina et al., 2019a, Soriano-Molina et al., 2019b, Soriano-Molina et al., 2021; Starling et al., 2021a). This reactor earns particular interest due to its higher treatment capacity and lower cost when compared to other reactors (Esteban García et al., 2018). Moreover, there are a plenty of studies for its application in a full scale, especially in the South of Spain (Maniakova et al., 2022, Maniakova et al., 2021; Miralles-Cuevas et al., 2021; Sánchez-Montes et al., 2022; Sánchez Pérez et al., 2020). Fig. 3 presents main photoreactors and information related to studies applied for the removal of pharmaceutical drugs present in MSE via solar photo-Fenton at pilot scale.

Studies shown in Table 1 aimed at post-treatment of MSE via solar photo-Fenton and were developed in various regions of the world including developed countries. Only one of the listed studies investigates the efficiency of photo-Fenton as post-treatment of MSE in a CPC, thus demonstrating the great interest of the scientific community in RPRs. Disregarding the photoreactor, 30 % of studies performed at pilot scale analyzed the removal of target-CECs (20–200 μg L^−1^) spiked to MSE due to analytical limitations associated to the detection of these compounds at reduced concentration levels. Furthermore, Table 1 shows that there is a scarcity of studies that apply solar photo-Fenton using Fe^2+^ at circumneutral pH and great interest in the use of chelating agents such as Fe^3+^-EDDS in developed countries. The main achievements and limitations encountered in these studies are analyzed in this review considering possible application of photo-Fenton as post-treatment of MSE generated from UASB and UASB + PT. A few studies carried out with synthetic wastewater matrix were also considered due to their relevance to this subject. Table 1 summarizes key findings achieved in each of these studies.

**Table 1:** Solar photo-Fenton performed in the post-treatment of secondary municipal wastewater treatment plant for the removal of pharmaceutical drugs.

CEC	Concentration	Matrix (treatment technology)	Reactor	Reactor features	Iron Source	pH	Experimental Scale	Results	Reference
Acetamiprid (ACTM) group of 77 CECs, mainly drugs, belonging to different therapeutic groups, including some of their metabolites.	ACTM: 200 μg L^−1^ Total CECs: 30.3 ± 0.3 μg L^−1^	SW and MSE (CAS)	RPR (fiberglass – SW, and PVC – MWW)	**SW**: 5 cm liquid depth, 120 L of total volume and a mixing time of 5 min. **MWW**: 80 L of total volume with 15 cm of liquid depth with a mixing time of 3 min, or 18 L of total volume, with a liquid depth of 5 cm and a mixing time of 2 min.	Fe^2+^ (5.5 mg L^−1^)	Acid 3.0	Pilot-scale	No significant difference for different liquid depths tested during continuous operation mode (87 % for 15 cm and 89 % for 5 cm). For the different HRT, removal rates were: 79 % (20 min), 84 % (40 min) and 89 % (80 min).	(Arzate et al., 2017)
77 CECs, mainly drugs, belonging to different therapeutic groups, including some of their metabolites	0.1 ng L^−1^ to 31 μg L^−1^	MSE (CAS)	RPR (PVC)	0.98 m of length and 0.36 m width, divided by a central wall. A paddlewheel at 200 rpm, mixing time of ~1.52 min.	Fe^2+^ (3 × 20 mg L^−1^)	Natural	Pilot-scale	99 % removal of total CECs load within 90 min of treatment. However, some compounds still showed resistance to oxidation (salicylic acid). 56 % of the DOC was removed during treatment, decreasing from 20.6 to 9.1 mg L^−1^.Total H_2_O_2_ consumption after 90 min.	(Freitas et al., 2017)
Sulfamethoxazole (SMX) as a model CEC and 116 CECs including drugs, belonging to different therapeutic groups.	SMX: 50 μg L^−1^. Total CECs: 14 to 44 μg L^−1^.	MSE (CAS)	RPR	**Lab. scale**: internal diameter = 10 cm; height = 20 cm. Lateral walls covered. **Pilot scale**: 19 L of total volume, channel width 22 cm and mixing time = 2 min.	Fe^2+^ (20 mg L^−1^) Fe^3+^-EDDS (5.6:58 mg L^−1^)	Natural	Laboratory and pilot-scale	**Lab. scale**: For the Fe^3+^-EDDS, decrease in the liquid depth increased SMX removal, leading to >70 % removal regardless of the liquid depth. **Pilot scale**: 34–45 CECs were detected in wastewater. 35 ± 11 % CECs removal achieved with FeSO_4_ and 81 ± 17 % with Fe^3+^-EDDS after 15 min of reaction.	(Soriano-Molina et al., 2021)
Diclofenac (DCF), Trimethoprim (TMP) and Carbamazepine (CBZ)	200 μg L^−1^ of each	UP and MSE (CAS)	CTC	Irradiated volume of 10.24 L, 8 acrylic glass tubes (150 cm length, 3.3 cm external diameter and 0.25 cm thickness).	Fe^2+^ (5.6 mg L^−1^) Fe^3+^-EDDS (5.6:58 mg L^−1^)	Natural	Pilot-scale	**Fe**^**2+**^**in MWW**: Dark Fenton (0 min) removed 50 % of DCF; photo-Fenton reached 85 % removal of DCF in 30 min, and 40 % CBZ removal in 120 min. **Fe**^**3+**^**-EDDS in MWW**: 99 % removal of all CECs in 15 min.	(Maniakova et al., 2020)
Naproxen (NAP)	226,36 μg L^−1^	UP and MSE (UASB + PT)	Solar reactor	Conventional glass recipient depth: 4.9 cm; diameter: 15.5 cm, irradiated surface: 189 cm^2^, total volume: 500 mL artificial irradiation by two black light lamps (10 W each, 350–400 nm) placed in parallel (3.5 cm) 1.0 cm above the surface	Fe^3+^-oxalate Fe^3+^-citrate Fe^3+^-NTA Fe^3+^-EDTA Fe^3+^-EDDS	7.0	Laboratory scale	Best results obtained in the presence of Fe/EDDS (1:1) and Fe/NTA (1:1), Fe/EDTA (1:2), Fe/Cit (1:3) and Fe/Ox (1:12). NAP removal from UP was faster when using H_2_O_2_ (20 kJ m^−2^ required) compared to S_2_O_8_^2−^ (90 kJ m^−2^ required). Better performance of S_2_O_8_^2−^ was observed in MWW. Fe^3+^-citrate complex with H_2_O_2_ was the most cost-effective alternative for both matrices.	(Silva et al., 2021a)
Caffeine (CAF), carbendazim (CBZ), and losartan potassium (LP)	100 μg L^−1^ each	MSE (CAS)	Solar reactor and RPR	**Lab. scale**: Conventional glass recipient (400 mL) under simulated solar radiation (SunTest CPS+). **RPR pilot-scale**: 1.22 m maximum length, 1.02 m central wall length and 0.20 m width; total volume of 12 L; 5 cm liquid depth	Fe^2+^ (5 × 11 mg L^−1^)	7.0	Laboratory and pilot scale	**Lab. scale**: the strategy to perform solar photo-Fenton at neutral pH using intermittent iron additions was effective for modified photo-Fenton using persulfate as oxidant. **Pilot scale**: final removal of CECs obtained during solar/Fe/H_2_O_2_ at near-neutral pH reached 49 % (2.5 kJ L^−1^). Solar/Fe/S_2_O_8_^2−^ showed higher efficiency of CECs removal (55 %) (1.9 kJ L^−1^).	(Starling et al., 2021a)
Nimesulide (NMD), furosemide (FRS), paracetamol (PCT), propranolol (PPN), dipyrone (DIP), fluoxetine (FXT), diazepam (DZP), and progesterone (PRG)	500 μg L^−1^ of each	UP and SW	Solar reactor	1 L of total volume in a bold bottom reflector.	Fe^3+^-EDDS (EDDS: 0.05, 0.28 and 0.50 mmol L^−1^)	Natural	Laboratory-scale	99.9 % and 98.7 % removal of total CECs from UP and SW, respectively.	(Cuervo Lumbaque et al., 2019)
Acetamiprid (ACTM)	100 μg L^−1^	SW	RPR (Cylindrical PVC)	**Lab scale**: Stirred tank reactors, 0.85 L total volume, 15 cm diameter, conducted in SunTest CPS. **Pilot scale**: RPR made of PVC, 19 L, liquid depth of 5 cm, channel width of 22 cm and mixing time of 2 min.	Fe^3+^-EDDS (0.1:0.1 mM)	Natural	Laboratory and Pilot-scale	**Lab scale**: In all cases, the model was accurate to reproduce experimental data regarding ACTM removal in the first 5 min of reaction and with good prediction until 10 min. **Pilot scale**: results are similar to those observed in the solar simulator. Nearly 80 % removal of ACTM in winter and spring.	(Soriano-Molina et al., 2018)
*O*-desmethyltramadol (O-DSMT), *O*-desmethylvenlafaxine (O-DSMV) and gabapentin (GBP)	O-DSMT: 0.32–1.98 μg L^1^ O-DSMV: 0.681.32 μg L^1^, GBP: 0.94–7.86 μg L^−1^	MSE (CAS)	RPR (PVC)	Liquid depth of 5 cm, volume of 19 L and length of the straight section of 2.4 m. For the liquid depth of 15 cm, volume of 78 L and length of the straight section of 5.1 m.	Fe^3+^-EDDS (5.6:29 mg L^−1^)	Natural	Pilot-Scale	Comparing kinetic constants obtained for CEC oxidation, differences between effluents are not causally related to salinity. In the continuous operation flow, 78 % (HRT 15 min) and 76 % (HRT 10 min) CECs removal were obtained in 5 cm and 15 cm depth in the RPR.	(Soriano-Molina et al., 2019b)
A group of 92 CEC, most of them pharmaceuticals and agrochemicals	Total load ranges from 6.2 to 58 μg L^−1^	MSE (CAS)	RPR (PVC)	Total volume of 19 L, liquid depth of 5 cm, channel width of 22 cm and mixing time of 2 min.	Fe^3+^-EDDS (5.6:29 mg L^−1^)	Natural	Pilot-Scale	No detrimental effect of the initial concentration of the main anions upon CECs removal. Average removal of CECs for the 5 MWW effluents from CAS was 83 % (15 min; 30 W m^−2^).	(Soriano-Molina et al., 2019a)
A group of 92 CEC, most of them pharmaceuticals and agrochemicals	Total load of 15.8 ± 81 μg L^−1^	MSE (CAS)	RPR (PVC)	Total volume of 16 L, length of 0.98 m and width of 0.38 m; liquid depth of 5 cm.	Fe^3+^-EDDS (5.6:29 mg L^−1^ 5.6:58 mg L^−1^)	Natural	Pilot-Scale	CEC removal for both Fe^3+^EDDS molar ratios tested in batch were similar: 65 % (1:1) and 69 % (1:2). In continuous treatment, CEC removal was 51 ± 3 % (1:1) and 57 ± 8 % (1:2).	(Arzate et al., 2020)
Acetaminophen, caffeine, carbamazepine, diclofenac, sulfamethoxazole and trimethoprim	**Lab scale**: 100 μg L^−1^ **Pilot scale**: 20 μg L^−1^	MSE (CAS)	RPR	**Lab. scale**: internal diameter of 19 cm and height of 19 cm. Liquid depth of 7 cm (1.6 L), 10 cm (2.4 L) and 15 cm (3.9 L). The lateral walls were covered. **Pilot scale**: volume of 19 L, channel width of 22 cm and. Reflexive aluminum surface at the bottom.	Fe^3+^-EDDS (Fe^3+^: 1, 2, 3, and 5.5 mg L^−1^)	Natural	Laboratory and Pilot-Scale	All experimental conditions achieved maximum efficiency for all the different liquid depths tested within 15 min of reaction. The positive influence of the reflexive surface on the bottom of the reactor was more apparent with lower initial iron concentrations.	(Costa et al., 2020)

Note: SW = simulated wastewater; MSE = municipal secondary effluent; UP = ultrapure water; CAS = conventional activated sludge; UASB + TP = Upflow anaerobic sludge blanket reactor followed by post-treatment; RPR = raceway pond reactor; CTC = compound triangular collector. Only Arzate et al. (2017) did not perform the treatment at circumneutral pH.

All studies summarized in Table 1 are related to application of solar photo-Fenton as post-treatment of CAS effluent aiming CEC removal, except for the analysis performed by Silva et al. (2021a) which analyzed CEC removal from an effluent sampled in the output of a UASB + PT. This confirms lack of data on the application of solar photo-Fenton as post-treatment of UASB effluents.

As shown in Fig. 2, there are significant differences among physicochemical properties of CAS, UASB, and UASB + PT effluents. Even though results obtained in studies shown in Table 1 are mostly relevant to the reality of developed countries and urbanized regions, limitations and tendencies found in these studies may guide process adaptation for its application as post-treatment of MSE from UASB reactors which are widely used for the treatment of domestic sewage in Latin America (ANA, 2017; Noyola et al., 2012).

A previous study compared three different photoreactors in the classic photo-Fenton process (FeSO_4_ at pH 2.8; intermittent additions of H_2_O_2_ during treatment): CPC, flat collector (FC), and RPR. The goal was to reach 75 % mineralization of four CECs (45, 90, 180 and 270 mg L^−1^ of DOC) (Cabrera-Reina et al., 2019). Among all tested reactors, the RPR required a longer treatment time to achieve target mineralization for both liquid depths. Even so, the economic evaluation presented for the treatment (mg DOC €^−1^ min^−1^; 10,000 m^2^, initial DOC of 90 mg L^−1^, and 15-cm liquid depth) using a RPR estimated a cost equivalent to 19.78 mg DOC removed €^−1^ min^−1^. This cost was significantly lower than costs calculated for tubular reactors (0.37 and 0.50 mg DOC removed €^−1^ min^−1^ for the CPC and the FC, respectively). Even though the RPR required a longer treatment time when compared to CPC and FC reactors, the authors concluded that the RPR is the best option for CEC removal as a lower area is necessary to treat the same volume of wastewater in this reactor compared with the other photoreactors. These findings emphasize the advantages of using RPR for the application of solar photo-Fenton as post-treatment of MSE.

Cabrera Reina et al. (2020) shed new light on the influence of temperature and sunlight availability and intensity upon solar photo-Fenton performance by comparing the efficiency of this process in three different countries (Spain, Chile, and Qatar) which receive relatively high incidence of solar irradiation, yet present different climate patterns. According to the study, in addition to incident solar irradiation, climate also plays an essential role in the efficiency of solar photo-Fenton, and reactor design should take both into account for each specific application and location. Moreover, this study shows a difference in solar reactor plant design for each season in a same location. Thus, ratifying the need to assess the performance of solar photo-Fenton in various seasons or to consider the worst-case scenario (winter season) related to incident irradiation, as pointed out elsewhere (Starling et al., 2021b). After all, considering the annual average sunlight irradiation for the design of a treatment plant based on a solar technology may underestimate the critical area required to reach target CECs removal during the winter, especially in temperate regions. Therefore, there is critical need for studies concerning the operation of solar photo-Fenton in continuous flow mode and simulating different scenarios to enable the application of the treatment throughout the year and in effluents generated from other biological treatment technologies, such as UASB and UASB + PT.

Solar photo-Fenton treatment was conducted in batch in most studies listed in Table 1. Yet, continuous operation mode aiming at the degradation of acetamiprid (ACTM) as a model contaminant on CAS effluent at pH 3.0 was studied in a RPR (Arzate et al., 2017). This study demonstrated the viability of operating solar photo-Fenton in continuous mode. Experiments were first performed on synthetic secondary wastewater (SW), then as post-treatment of MSE. Different hydraulic residence times (HRT) and liquid depths were tested to optimize the removal of CECs, thus raising essential background knowledge. Considering results reported in the referred study and continuous generation of MSE in a treatment facility, it is better to work at increased depths to ensure maximum treatment capacity. Minimum CECs removal corresponded to 79 % and was observed at a HRT equivalent to 20 min. The study confirmed the feasibility of applying continuous photo-Fenton treatment at low HRT which increases chances of using this process on a full-scale. However, continuous operation is not sufficient to enable full-scale application of photo-Fenton as it is also essential to consider limitations related to optimum pH (≈2.8) for photo-Fenton operation.

### 3.2 Photo-Fenton operation at neutral pH: complexing agents and intermittent iron additions

Considering higher solubility of iron at acidic pH, the optimum pH range for the operation of Fenton treatments is between 2.8 and 3.0 (Tarr, 2003). However, natural matrices, such as MSE usually present neutral pH and pH adjustment increases operational costs and wastewater salinity after treatment, apart from risks associated to storage of large volumes of acid and base before and after treatment, respectively. The use of iron complexing agents has been stimulated to overcome this limitation, as these complexes increase iron solubility at near-neutral pH (Costa et al., 2021a; Maniakova et al., 2022, Maniakova et al., 2021; Miralles-Cuevas et al., 2021; Sánchez-Montes et al., 2022). Soriano-Molina et al. (2021) compared the performance of photo-Fenton treatment using Fe^3+^-EDDS complex with Fe^2+^ as iron sources at circumneutral pH in a pilot-scale (RPR). A maximum of 35 % CECs removal was achieved for Fe^2+^ while Fe^3+^-EDDS reached 81 % removal. This indicates that it is more effective to use Fe-complex as a source of iron for the treatment of MSE at neutral pH compared to Fe^2+^.

In this regard, Maniakova et al. (2020) compared Fe^2+^ with Fe^3+^-EDDS as iron sources for the removal of diclofenac (DFC), trimethoprim (TMP), and carbamazepine (CBZ) from ultrapure water (UP) and MSE via photo-Fenton treatment at circumneutral pH in a compound triangular collector (CTC). While 99 % removal of all CECs was obtained in the presence of Fe^2+^ in UP within 4 min by the dark Fenton process, a maximum of 50 % TMP degradation was achieved by dark Fenton in MSE after 120 min. In contrast, removal of target CECs by photo-Fenton in the real matrix was limited to 58 % (120 min, Q_UV_ = 12.1 kJ L^−1^). These results confirm the influence of matrix composition upon CEC removal via Fenton processes. The use of EDDS as a chelating agent improved solar photo-Fenton efficiency, leading to 99 % removal of CECs from MSE within 15 min (Q_UV_ = 1.2 kJ L^−1^). Although chelating agents have not yet been reported for the removal of CECs from UASB systems effluents, these results indicate the use of complexing agents as an appropriate strategy to conduct photo-Fenton at neutral pH. However, considering that UASB systems are used for municipal sewage treatment mainly in developing countries, costs associated to the addition of complexing agents may be a significant drawback in this region.

A recent work has evaluated the use of complexing agents to remove naproxen from effluent sampled in the output of a MWWTP which applies UASB + PT (coagulation-flocculation-ferric chloride, FeCl_3_ – and flotation) (Silva et al., 2021a). The study compared the performance of photo-Fenton using different complexing agents (Fe^3+^-oxalate (FeOx), Fe^3+^-citrate (FeCit), Fe^3+^-nitrilotriacetic acid (FeNTA), Fe^3+^-ethylenediaminetetraacetic acid (Fe-EDTA) and Fe^3+^-EDDS and oxidants (H_2_O_2_ or S_2_O_8_^2−^)). Treatment efficiency was strongly dependent on the Fe-complex, iron/ligand molar ratio, oxidant agent, and matrix composition. The work demonstrated that S_2_O_8_^2−^ is more efficient than H_2_O_2_ in treating UASB + PT effluent, probably due to higher selectivity and lifespan associated to sulfate radical compared to HO• (Duan et al., 2020; Wacławek et al., 2017). However, considering high commercial prices of persulfate, the option which gave the best cost-benefit was the treatment performed using FeCit + H_2_O_2_.

Still regarding the use of Fe^3+^-EDDS complex in the photo-Fenton treatment, Fe^2+^ concentration was only stable for the 1:2.5 Fe:EDDS molar ratio. This indicates that an excess of EDDS is necessary to maintain this catalyzer available during the photo-Fenton reaction applied as post-treatment of MSE. The chelating complex remained stable in the system for 120 min (Cuervo Lumbaque et al., 2019). This fact must be considered to define the most appropriate HRT to be applied for post-treatment of MSE, especially when aiming at continuous operation of solar photo-Fenton treatment in real treatment plants. In fact, some studies have proved that the removal of pharmaceutical drugs from MSE generated by aerobic systems via solar photo-Fenton occurs at the beginning of treatment indicating that photoreactor scale-up aiming at a minimum of 80 % CECs removal may consider HRT ranging from 10 to 30 min (Soriano-Molina et al., 2019b). However, as UASB and UASB + PT effluents are more complex than CAS effluent, HRT required for the removal of CECs from these matrices may be longer.

A mechanistic model for the removal of ACTM via solar photo-Fenton conducted at neutral pH in SW in the presence of Fe^3+^-EDDS and as a function of radiance and reactor geometry was developed by Soriano-Molina et al. (2018). As suggested in the model, UV light promotes the conversion of soluble Fe^3+^-EDDS into Fe^2+^-EDDS. Kinetic models for laboratory-scale experiments showed that the oxidation of Fe^2+^-EDDS by H_2_O_2_ (k = 1.9 ∙ 10^3^ mM^−1^ min^−1^) is three orders of magnitude higher than the reaction between Fe^2+^ and H_2_O_2_ which occurs in the classic Fenton reaction (k = 4.6 mM^−1^ min^−1^) (Buxton et al., 1988). In contrast, the reaction between Fe^2+^ and H_2_O_2_ in the classic Fenton reaction generates Fe^3+^, which precipitates at neutral pH. This explains higher H_2_O_2_ consumption within the first few minutes of reaction when EDDS is used as a complexing agent. In addition, Fe hydroxides formed in the classic system showed lower light absorption when compared to Fe^3+^-EDDS. Longer lifespan of soluble iron after its decomposition in the process conducted in the presence of EDDS is advantageous, and may be attributed to the presence of Fe^3+^-EDDS oxidized species generated by its reaction with HO• (Huang et al., 2013). Experiments performed under real solar radiation in a RPR confirmed data adjustment to the model with regard to H_2_O_2_ consumption, total dissolved iron profile, Fe^3+^-EDDS decomposition, and ACTM degradation. The most important contribution of the referred study is related to the applicability of the kinetics model developed with data obtained in laboratory-scale for data obtained in large scale using RPR. Therefore, this kinetic model can be useful for solar photo-Fenton scale-up for application in real MWWTP.

Nonetheless, attention should be given to matrix composition. Model performance (Soriano-Molina et al., 2018) was evaluated for the treatment of CAS effluent samples from 5 MWWTP (Soriano-Molina et al., 2019a). The target was to maximize treatment capacity in terms of volume of MSE treated per unit of reactor surface area and time (L m^−2^ d^−1^). Three model drugs were tracked during experiments: *o*-desmethyltramadol (O-DSMT), *o*-desmethylvenlafaxine (O-DSMV), and gabapentin (GBP). The kinetic model successfully predicted reagent consumption and CECs degradation (76 % in 10 min, 78 % in 15 min, 5 cm of liquid depth). Yet, Fe^3+^-EDDS conversion was slightly overestimated for a liquid depth of 15 cm. Even so, the model successfully predicted H_2_O_2_ consumption and CECs removal for all effluents. Differences regarding the removal of CECs in each effluent were associated to the composition of effluent organic matter as it affects iron availability in the system. Considering higher concentration of organic matter in MSE from UASB systems compared to those from CAS systems, it is imperative to analyze the applicability of this model for post-treatment of anaerobic effluents.

Although organic matter present in MSE may hinder treatment efficiency, the use of NOM such as humic acid and humic like substance as chelating agents to enable photo-Fenton operation at neutral pH has also been studied in the past few years. According to Georgi et al. (2007), NOM are classified into humic acid, fulvic acid or humin as according to their solubility. Humic acid is insoluble at pH < 2, while fulvic acid is soluble at all pH values. Overall, humic acid molecules are larger than fulvic acids and contain more aromatic rings with a large number of attached —OH and —COOH groups, while fulvic acids hold a higher number of carbonyl and aliphatic groups (Lindsey and Tarr, 2000). These substances are known to form complexes with metal ions as they bind to carboxylate, polyphenolic and nitrogen-containing sites. The presence of carboxylic, phenolic, alcoholic, quinone, amino and amido groups enables ionic exchange, complex formation and oxidation-reduction processes (Zhang and Zhou, 2019). In this sense, special interest has been given to wastewaters generated from industrial processes and which could be a potential source of NOM to be used as iron complexing agents, such as olive mill wastewater. However, comparable removal of CECs using NOM or Fe^3+^-EDDS (>80 %) as complexing agents were only reached at pH 5 (Davididou et al., 2019; García-Ballesteros et al., 2018; Ruíz-Delgado et al., 2019). Prada-Vásquez et al. (2021) presented strategies for the elimination of CECs via photo-Fenton process using Ibero-American effluents as NOM sources. Despite promising results in the use NOM present in wastewaters as iron-complexes to promote the photo-Fenton processes, their concentration in these matrices was limited to a few mg L^−1^, thus showing limited practical application for post-treatment of MSE in real scale. In addition, as there is no data regarding the use of NOM-rich wastewaters as alternative sources of chelating agents for solar photo-Fenton treatment of UASB effluents, this could be an interesting approach for future research.

Despite inhibitory effects promoted by matrix constituents during oxidative treatments (Lado Ribeiro et al., 2019), Soriano-Molina et al. (2019b) demonstrated no detrimental impact upon reaction rate or CECs removal for different initial concentrations of the main cations and anions among CAS effluents from 5 MWWTP. Thus, the authors indicated that organic matter present in MSE is the main disturbing factor for the removal of CECs under similar treatment conditions. Although most studies perform solar photo-Fenton as post-treatment of CAS effluent, other technologies (anaerobic reactors, membrane bioreactors and moving bed biological reactors) are also applied in MWWTPs and each of these technologies lead to distinct organic matter composition in MSE. Hence, solar photo-Fenton treatment conditions must be adapted mainly to the concentration of organic matter present in each MSE before its application as a tertiary treatment in real MWWTPs. Considering NOM content in MSE generated by each treatment technology, it is expected that minimum accumulated UV energy and required oxidant concentrations may follow the following order from higher to lower: UASB effluent > UASB + PT effluent > CAS effluent (Fig. 2E, F).

Arzate et al. (2020) were the pioneers to test the operational feasibility of photo-Fenton using EDDS as a chelating agent in continuous flow for three consecutive days aiming at post-treatment of MSE. Results demonstrated that percentage of CECs removal were reasonably similar for Fe^3+^:EDDS molar ratios equivalent to 1:1 and 1:2. Despite the difference in the total load of CECs (15.8–77.5 μg L^−1^) in each MSE sample, CECs removal ranged between 51 ± 3 % (20 min) and 56 ± 4 % (40 min). Similar profiles of CECs removal obtained for all samples may be associated to comparable organic matter content (DOC 14.0–22.5 mg L^−1^). Global degradation of CECs was equivalent to 66–67 % during continuous operation (HRT 20 min and Fe^3+^:EDDS 1:1) (Soriano-Molina et al., 2021).

The conception and design of a treatment plant should not only consider the capacity of the AOP to mineralize CECs. Yet, it should also account for the natural variability of solar radiance throughout the year and the incident irradiation particularities for each region. One of the options to overcome natural solar irradiation shifts is the use of Light Emitting Diode (LED) lamps for cloudy/winter days (Silva et al., 2021b). Another strategy tested to enhance photo-Fenton treatment in a RPR is to improve the optical pathway by installing a reflexive aluminum surface at the bottom of the reactor (Costa et al., 2020). This adaptation was positive for low initial iron-EDDS concentrations (0.018 mM and 0.036 mM), as higher iron concentrations increased wastewater turbidity at neutral pH, thus reducing the probability of photo-activation of the iron catalyst. Strategies such as the use of a reflexive surface to enhance light absorbance may enable the application of this process for post-treatment of UASB effluents as they present higher TSS and turbidity (Fig. 2C, D) which may scatter light reducing treatment effectiveness.

Despite various studies with chelating agents, the use of these agents adds organic matter to the matrix, increases costs, and has unknown toxicity (Ahile et al., 2020b). Thus, Carra et al. (2013) proposed the use of the intermittent iron addition strategy to overcome these limitations and enable photo-Fenton operation at neutral pH at lower costs. The strategy was further tested by Freitas et al. (2017) for post-treatment of MSE via photo-Fenton treatment in a RPR. The authors observed pH decay after each Fe^2+^ addition which contributed to the continuous presence of dissolved iron in aqueous solution and, consequently, to the occurrence of photo-Fenton reactions, thus leading to 99 % removal of CECs within 90 min of treatment. Iron additions occurred at 5, 15, and 20 min, yet this procedure required two times more FeSO_4_ than the amount applied by Soriano-Molina et al. (2021).

Starling et al., 2021a, Starling et al., 2021b studied the use of S_2_O_8_^2−^ as an alternative oxidant to perform solar photo-Fenton for post-treatment of CAS effluent at neutral pH in a pilot-scale RPR aiming at the degradation of caffeine (CAF), carbendazim (CBZ), and losartan potassium (LP) (100 μg L^−1^ each). This was the first study to test and confirm the efficiency of the intermittent iron addition strategy using S_2_O_8_^2−^ as an oxidant and higher removal of CECs was obtained when using S_2_O_8_^2−^ (55 %; 1.9 kJ L^−1^) compared to H_2_O_2_ (49 %; 2.5 kJ L^−1^). In contrast to Silva et al. (2021a), the study showed that the total cost (operational costs + investment costs + amortization costs) associated with the modified solar photo-Fenton process in the presence of S_2_O_8_^2−^ (0.6 € m^−3^) was lower when compared to traditional solar photo-Fenton (H_2_O_2_; 1.2 € m^−3^). In this case, the lower cost associated with treatment performance using persulfate was highly influenced by the reduced surface area required for this oxidant. Performing cost evaluations is essential to choose the most appropriate post-treatment strategy, especially for complex matrices such as UASB and UASB + PT effluents.

### 3.3 Transformation products formed during post-treatment of MSE treatment

Another key issue which must be investigated during solar photo-Fenton application as post-treatment of MSE is the identification of by-products formed from multiple and simultaneous degradation reactions occurring during treatment. The identification of by-products elucidates the pathway of degradation of each CEC during the oxidative process. The application of solar photo-Fenton to degrade six drugs (dipyrone, diazepam, fluoxetine, acetaminophen, propranolol, and progesterone at 500 μg L^−1^) at circumneutral pH in wastewater using Fe^3+^-EDDS (1:1 and 1:2, 15.35 mg L^−1^ of iron and 230 mg L^−1^ of H_2_O_2_) reached high degradation levels (>77 %; t_30W_ = 57 min) (Cuervo Lumbaque et al., 2021a). The authors reported the identification of 21 by-products from the oxidation of target drugs during treatment, of which 16 were still present in samples after treatment.

Also, Sanabria et al. (2021) studied the degradation of anastrozole (50 μg L^−1^) in aquatic matrices via solar photo-Fenton process. Solar photo-Fenton removed 95 % of anastrozole in deionized water (t_30w_ = 65.8 min, 5 mg Fe^2+^ L^−1^, 25 mg H_2_O_2_ L^−1^, pH 5.0), while the treatment performance in real wastewater was limited to 51 % (intermittent additions of iron at 10 mg Fe^2+^ L^−1^, 25 mg H_2_O_2_ L^−1^, pH 5.0). Five by-products of anastrozole were identified in deionized water and only two of them were also detected in wastewater. The main degradation pathways proposed by authors were demethylation and hydroxylation. The lower number of by-products detected in real wastewater is probably associated to the lower efficiency of the solar photo-Fenton process. In real matrices, NOM and inorganic substances may act as HO• scavengers and/or compete with target compounds, thus decreasing treatment efficiency (Maniakova et al., 2021; Rizzo et al., 2020).

As UASB effluent presents higher organic matter content than CAS effluent, the degradation of target CECs may be limited in this matrix resulting in a reduced number of by-products during treatment. Due to lack of studies exploring the treatment of this matrix it is not possible to conclude what are the consequences of matrix composition to degradation mechanisms occurring in this matrix. This calls attention to the critical need for research on this direction.

## 4 Toxicity evolution during solar photo-Fenton as post-treatment of municipal wastewater treatment plant effluent at pilot-scale

One of the leading environmental concerns associated with the presence of pharmaceutical drugs in MSE is effluent toxicity (Dalgaard, 2012) as these compound may cause effects to aquatic biota even when in low concentrations (μg L^−1^ to ng L^−1^). Hence, the strategy of the European Union (EU) to preserve ecosystem integrity involves of the removal of CECs from MSE via post-treatment. The EU has recently invested in programs that stimulate research and development of technologies that enable the removal of pharmaceuticals and antimicrobial-resistance bacteria and genes from treated wastewater before disposal in surface waters (European Commission, 2020).

The 2008/105/EC Directive of the European Parliament and the Council imposed (i) the identification of all causes of chemical pollution of surface water representing a threat to the aquatic environment (acute and chronic toxicity to aquatic organisms) and (ii) implementation of pollution control measures at the source. Also, the EU Watch List included metaflumizone, amoxicillin, ciprofloxacin, sulfamethoxazole, trimethoprim, venlafaxine and *O*-desmethylvenlafaxine, dimoxystrobin, famoxadone, and 10 azole compounds to the list of substances for Union-Wide monitoring after careful revision by the Commission Implementing Decision EU/2020/1161. This selection considered the toxicity of listed substances along with sensitivity, reliability, and comparability of monitoring methods.

In addition to analytical methods that allow for monitoring of listed compounds, standardized bioassays (OECD, ISO) using different test-organisms allow for the quantification of toxic effects promoted environmental samples, such as MSE, to different trophic levels. Bacteria (e.g., *Aliivibrio fischeri* and *Photobacterium phosphoreum*), invertebrates (e.g., *Daphnia magna*), algae (e.g., *Chlorella vulgaris* and *Raphidocelis subcapitata*), fish (e.g., *Danio rerio*), and plants (e.g., *Lactuca sativa* and *Sinapis alba*) are currently used as test-organisms for this purpose. Tests sold as kits such as the Microtox bioassay (MICROTOX®), Medaka multigeneration Test (MTT), *Tetrahymena thermophila* bioassay (PROTOXKIT F), and bacterial luminescence test (BLT) are some of the main tests used to assess toxicity of water and wastewater samples (Tufail et al., 2020).

Table 2 presents CECs covered in the European Union's latest Watch List and toxicity data for these compounds (ECOTOX, 2022) considering acute toxicity to *Daphnia magna* (consumer) and chronic toxicity to *Raphidocelis subcapitata* (primary producer). Details on toxicity data, such as endpoints selection criteria and exposure period, is described in Rico et al. (2019). As shown in Table 1, EC_50_ values reported for *R. subcapitata* (chronic toxicity) are bellow EC_50_ values reported for acute toxicity with *D. magna*, and non-effect concentration (NOEC) values for all substances are in the range of 20 μg L^−1^.

**Table 2:** CECs enlisted in the European Union's Watch List and their acute and chronic toxicity responses.

Name of substance	Maximum acceptable method LOD (ng L^−1^)	*Daphnia magna*		*Raphidocelis subcapitata*	
		EC_50_ (mg L^−1^)	NOEC (mg L^−1^)	EC_50_ (mg L^−1^)	NOEC (mg L^−1^)
Metaflumizone	65	2.56	1.1	>0.31	0.31
Amoxicillin	78	>1500.00	ND	>1000	ND
Ciprofloxacin	89	1.20	10	18.70	<5.00
Sulfamethoxazole	100	96.70	ND	0.52	<0.50
Trimethoprim	100	92.00	ND	83.80	16.00
Venlafaxine	6	141.28	ND	ND	ND
*O*-desmethylvenlafaxine	6	ND	ND	ND	ND
Clotrimazole	20	0.21	ND	ND	ND
Fluconazole	250	ND	ND	ND	3.07
Imazalil	800	2.02	>1.80	0.73	ND
Ipconazole	44	1.70	0.13	ND	ND
Metconazole	29	ND	ND	ND	ND
Miconazole	200	ND	ND	ND	ND
Penconazole	1700	3.69	ND	ND	ND
Prochloraz	161	3.01	ND	ND	ND
Tebuconazole	240	4.00	0.49	2.73	1.19
Tetraconazole	1900	2.63	0.48	15.00	ND
Dimoxystrobin	32	ND	ND	ND	ND
Famoxadone	8.5	0.01	0.03	0.03	0.02

Note: LOD = limit of detection; ND = no data.

The implementation of this Watch List in developing countries such as those in Latin America, is a massive challenge as advanced analytical methods and sample preparation techniques (in example: Solid Phase Extraction, SPE) are required for their analysis by High Performance Liquid Chromatography coupled to Mass Spectrometry (LC-MS-MS). Although these methods are able to detect these analytes in low concentrations (low LOD), they may be expensive for developing countries. In addition, occurrence of CECs in the environment does not directly reflect ecological effect upon aquatic biota as some CEC may promote toxicity even below quantification and detection limits and simultaneous occurrence of a mixture of compounds in complex matrixes, such as MSE, may promote higher or lower toxicity due to synergism or antagonism, respectively. Therefore, CEC monitoring must be performed along with toxicity assays.

Additionally, when it comes to ecotoxicity, the European Environment Agency (EEA) report No. 23/2018 lists MWWTPs as significant contributors to direct releases of all groups of pollutants into water. The chemical industry and energy supply facilities are also substantial contributors to the release of substances that promote ecotoxicity to water and indirect releases of these substances to MWWTPs (M. Granger et al., 2019). Besides, the commission of evaluation of MWWTP recognized CECs as an environmental issue, and supports the upgrade of MWWTP with post-treatment stages aiming at toxicity removal (European Commission, 2020). In this regard, the Swedish Environmental Protection Agency Report No. 6766 presented AOPs as effective post-treatment alternatives to be implemented among available advanced technologies (Sundin et al., 2017).

Regarding the evaluation of toxicity during the application of AOPs as post-treatment of MSE, different reposes have been detected: i) toxicity reduction during AOP treatment, as observed for the photo-Fenton performed under artificial radiation and for the Fenton process; ii) toxicity increase in the beginning of treatment followed by decrease; iii) toxicity increase after AOP treatment as seen for the solar photo-Fenton (Wang and Wang, 2021). Toxicity responses after AOPs may be related to many factors, such as: types of reactive species active in the system, structure and chemical properties of organic pollutants (K_ow_, pka, solubility, among others) present in the matrix, concentration of reactive species, the toxicity assay selected to analyze matrix effect, experimental parameters, residual oxidants, and applied catalysts. It is known that partial oxidation of organic contaminants during treatment by AOPs can generate by-products which are more toxic than their parent molecule leading to increased toxicity (Oller and Malato, 2021; Rizzo, 2011).

The application of analytical tools based on high-performance liquid chromatography to monitor and quantify CECs, their transformation products and metabolites in the environment (Pinasseau et al., 2019) is highly challenging when it comes to MSE due to matrix complexity (Hinojosa Guerra et al., 2019). Analytical methods used for the surveillance of CEC and their degradation products in this matrix may show increased LOD and LOQ values (Rizzo et al., 2020, Rizzo et al., 2019). This is critical as some compounds may still promote toxic effects even when present at concentrations levels below LOD (Oller and Malato, 2021). Hence, bioassays should be applied along with analytical monitoring to detect acute and chronic toxic effects upon aquatic biota. In addition, bioassays enable the assessment of the impact promoted by interactions between a mixture of compounds (matrix components) and test-organisms, as it usually occurs in MSE and surface water after MSE disposal.

One alternative used to quantify individual risks associated to each compound detected in the environment is the ecotoxicological risk assessment (ERA). This method associates environmental concentration of a compound with safe concentration values defined for each substance as according to results obtained in ecotoxicological assays. In ERA, measured environmental concentration (MEC) obtained for each pollutant is divided by predicted no effect concentration (PNEC) obtaining a Risk Quotient (RQ). PNEC values are calculated by dividing acute (concentration which causes an effect to 50 % of the population, EC_50_) or chronic effect concentrations (non-observed effect concentration, NOEC) by an assessment factor (AF) which varies from 10 to 1000. Hence, if MEC is lower than PNEC there is no risk associated to the occurrence of that compound in the environment (RQ < 1). Whereas, if MEC is equal to or higher than PNEC (RQ ≥ 1) that compound presents an ecotoxicological risk. Gosset et al. (2021) investigated ecotoxicological risk concerning 41 CECs (37 pharmaceuticals and 4 pesticides) detected in 10 MWWTPs in France by evaluating the single CECs approach and the mixture of these substances. RQ were calculated according to Perrodin et al. (2011) based on the comparison between the predicted environmental concentration (PEC) of a compound or its mixture in water courses and its PNEC data obtained from ecotoxicity responses (1 < RQ < 10 = low risk; 10 < RQ < 100 = medium risk; 100 < RQ = high risk). 19 compounds (17 pharmaceutical drugs) detected in samples represented significant risks: *N*,*N*-diéthyl-3-méthylbenzamide (DEET) (RQ = 39.84), diclofenac (RQ = 62,10), lidocaine (RQ = 125.58), atenolol (RQ = 179.11), terbutryn (RQ = 348.24), atorvastatin (RQ = 509.27), methocarbamol (RQ = 1509.71), and venlafaxine (RQ = 3097.37). Although some CECs led to a negligible risk when evaluated individually, a significant risk was detected for the mixture. This fact must be considered for the selection of post-treatment technologies to be applied for MSE improvement, as CEC removal and organic matter composition may vary as according to the technology employed in each treatment plant.

A recent study performed a risk assessment of pharmaceutical drugs in surface waters in Brazil (Sodré et al., 2018). The authors selected MEC and PNEC values reported in literature for the main CECs investigated in the country. According to the study, pharmaceutical drugs trimethoprim, sulfamethoxazole, tetracycline, azithromycin, norfloxacin, acetaminophen, naproxen, diclofenac, caffeine, ciprofloxacin, sertraline, and carbamazepine exceed the trigger value for high risk (RQ > 1). Indeed, RQ obtained for some of these drugs (17α-ethynylestradiol, trimethoprim, sulfamethoxazole, diclofenac, caffeine, carbamazepine among others) was higher than 10,000. Moreover, Wielens Becker et al. (2020) investigated pharmaceuticals drugs and their metabolites in hospital wastewater in Brazil. RQ calculated for drug mixtures and their metabolites indicates high risk to aquatic organisms (*Green algae*, *Daphnid* and *Fish*). As direct discharge of hospital wastewater to municipal wastewater network or to surface waters are frequent practices in developing countries due lack of sanitation infrastructure (Azuma et al., 2019), results obtained in this risk assessment are very concerning (Marson et al., 2022), especially with regard to drugs and their metabolites.

Ideally, the selection of the best available technology for post-treatment of MSE generated by different methods should be based on recognition of main constituents/fractions responsible for MSE toxicity. This knowledge may be earned by the whole effluent toxicity (WET) method developed by the US Environmental Protection Agency (40 CFR 136.6) (eCFR). WET assay exposes test organisms to different fractions of the target wastewater and in different dilutions. WET results are used by the National Pollutant Discharge Elimination System (NPDES) to determine which is the fraction responsible for wastewater toxicity and whether the facility complies with environmental standards (US EPA, n.d.). According to Furley et al. (2018), one of the significant issues to be faced by Latin America (LA) countries relies on lack of adequate wastewater treatment. This problem must be solved as untreated wastewater is currently discharged into rivers, lakes, underground aquifers, and the ocean in this region. Overall, there is no specific directive or legislation regarding the toxicity promoted by urban wastewater to the aquatic ecosystem in this region. So, validation and implementation of WET evaluation using species native to LA would allow for the selection of the most appropriate technology to be used as post-treatment in this region.

Despite of lack of regulation regarding wastewater discharge toxicity in LA, Brazilian resolution CONAMA 430/2011 states that the effluent to be discharged onto surface water must not cause or have the potential to cause toxic effects to aquatic organisms (at least two different trophic levels) (Brazil, 2011). Still, lack of definition of specific assays for each effluent type compromises standardization. In addition, surface water quality standards impose absence of chronic toxicity in streams designated as high-quality waters (Classes 1 and 2) and absence of acute toxicity for streams classified as average quality waters (Class 3) (Brazil, 2005). These legal requirements reinforce the need for toxicity evaluation during post-treatment of MSE.

Several by-products are generated during the oxidation of both CECs and dissolved organic matter (DOM) present in MSE via AOPs (Maniakova et al., 2020), including solar photo-Fenton (Michael et al., 2019). These transformation products (TPs) may be even more toxic than parent compounds (Oller and Malato, 2021). Unknown TPs may be detected by Liquid chromatography coupled to mass spectrometry (LC-MS) even when at trace levels (Martínez-Piernas et al., 2021). However, due to lack of a commercial analytical grade standard to obtain a reference spectra in MS/MS for many TPs, some studies analyze the fragmentation-degradation relationship to elucidate TP structure (Wang et al., 2020). According to Cuervo Lumbaque et al., 2021a, Cuervo Lumbaque et al., 2021b, the identification of TPs is key to understanding how the degradation of CECs takes place in the solar photo-Fenton processes and indicates their environmental fate.

**Fig. 4:**
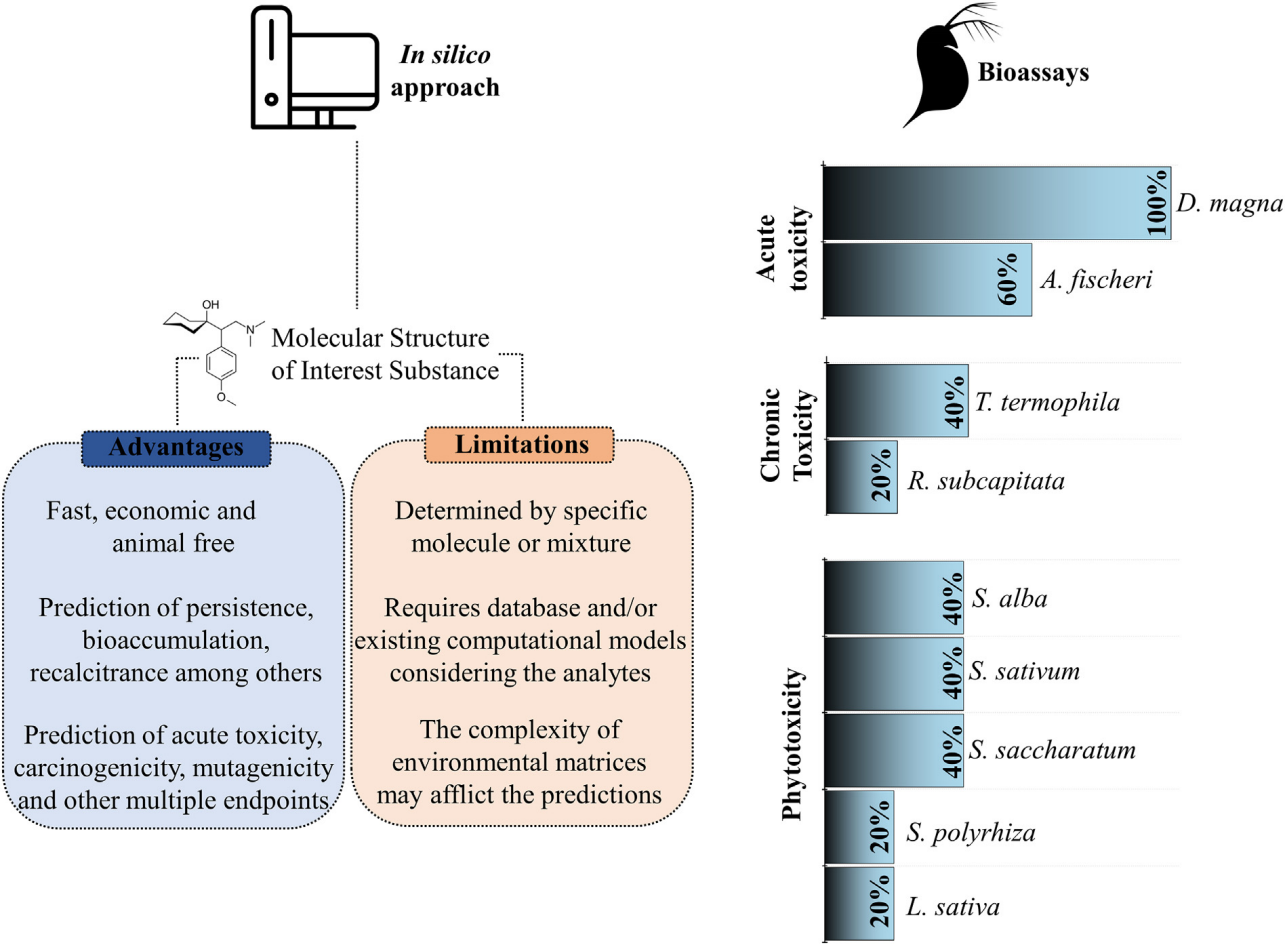
Advantages and limitations associated to in silico approach for toxicity evaluation and bioassays used to monitor the toxicity of municipal secondary wastewater during solar photo-Fenton.

TP structure may also be explored by in silico tools which enable toxicity prediction. Quantitative Structure-Activity Relationship (QSARs) consist of computational models that predict the toxicological behavior of detected compounds based on their physicochemical properties (Pizzo et al., 2013). Della-Flora et al. (2020) assessed the environmental fate and ecotoxicity of anticancer drug flutamide and TPs generated during the degradation of this compound via solar photo-Fenton (pH 5.0; Fe^2+^ 5 mg L^−1^; H_2_O_2_ 50 mg L^−1^) using in silico QSARs predictions. QSAR predictions linked detected TPs to mutagenicity and carcinogenicity effects which were confirmed by software analysis based on structural signs. Other studies evaluated the QSARs of CECs and TPs formed during solar photo-Fenton treatment of hospital wastewater (Cuervo Lumbaque et al., 2021b) in different aqueous matrices (Sanabria et al., 2021). Thus, QSARs may be a great tool to predict the toxicity of target CECs and by-products formed during post-treatment and to support the selection of the most appropriate ecotoxicological assays and post-treatment technology for each context. Advantages and limitations associated to in silico tools and percent use of bioassays applied for ecotoxicological assessment of MSE post-treatment by solar photo-Fenton are shown in Fig. 4.

In silico approaches used for toxicity evaluation before and after AOPs rise as a valuable tool in the absence of sufficient experimental data mainly because they are fast, economic and animal-free. Van Dijk et al. (2021) pointed out that this strategy may be the key to complementary toxicological studies concerning the screening and prioritization of toxic CECs present in the environment. In addition, they support decision-making on the selection of the most appropriate ecotoxicological assays as they predict potential hazards according to CECs chemical structure and physicochemical properties (Cassani and Gramatica, 2015). However, computational models for thousands of individual substances with known molecular structures and ecological effects allow for the prediction of persistence, bioaccumulation, and toxicity, thus being complimentary to threshold values established in regulatory guidelines. Still, these predictions may not effectively evaluate the effect CEC mixtures (Muir et al., 2019) as they are still based on the evaluation of single compounds. Hence, *in silico* tools should not replace bioassays, yet complement these analyses.

After all, bioassays offer vital insight into the feasibility of post-treatment alternatives for MSE treatment as they enable the detection of effects promoted by the mixture of compounds (Escher et al., 2020). In addition, bioassays are generally quick, simple, and low-cost (Lashuk and Yargeau, 2021). Fig. 4 shows that 100 % of studies (33 % of the total studies presented in Section 3, Table 1) which assessed toxicity of MSE treated by solar photo-Fenton upon aquatic organisms employed *D. magna* bioassay (acute toxicity). It can also be observed that there is a lower concern regarding the detection of chronic effects and phytotoxicity responses after treatment. Both tests are critical, as the concentration of CECs and their TPs in environmental compartments usually contribute to sublethal effects in the environment, and there is increasing tendency for wastewater reuse in irrigation.

Table 3 lists studies that evaluated the evolution of toxicity during solar photo-Fenton applied as post-treatment of MSE. It is important to emphasize the scarcity of data regarding toxicity of CAS effluent treated by solar photo-Fenton, and no information available for UASB or UASB + PT effluents. Michael et al. (2012) were the first to report phytotoxicity of samples obtained after solar photo-Fenton as post-treatment of MSE and to contrast responses with the removal of both CECs and DOM. The authors showed that toxicity responses in treated wastewater spiked with antibiotics were similar to those observed when organisms were exposed to treated wastewater samples alone, thus indicating that DOM contributes to sample toxicity. This is an important finding considering the application of solar photo-Fenton for UASB and UASB + PT effluents as organic matter content in these effluents is significantly higher when compared to CAS effluent (Fig. 2E, F).

**Table 3:** Toxicity evaluation during post-treatment of municipal wastewater treatment plant effluent by solar photo-Fenton at pilot-scale.

Test-Organism	Matrix (treatment technology effluent)	Reactor	Reactor features	Iron source	pH	Experimental scale	Results	Reference
*Daphnia magna* (acute toxicity), *Sinapis alba*, *Lepidium sativum*, and *Sorghum saccharatum* (phytotoxicity)	MSE (CAS)	CPC	Twelve borosilicate glass tubes with diameter of 55 mm and mounted on curved polished aluminum reflectors. The overall volume capacity of the reactor was 250 L and total irradiated volume was 85.4 L	Fe^2+^ (5 mg L^−1^)	2.8	Pilot scale	Treated samples (180 min) were more toxic than CAS effluent for *D. magna*. Toxic effect only decreased (6.7 %) after 300 min. The three plant species used on phytotoxicity assays displayed an interesting profile concerning germination, root and shoot inhibition stimulated by the presence of oxidation by-products. Solar photo-Fenton reduced phytotoxicity.	(Michael et al., 2012)
*Daphnia magna* (acute toxicity), *Sinapis alba*, *Lepidium sativum*, and *Sorghum saccharatum* (phytotoxicity)	MSE (CAS)	CPC	Total volume of 100 L with irradiated volume of 21.4 L. Glass tubes mounted on a fixed platform tilted at the local latitude (35°), reflecting surface was made of resistant and highly reflecting polished aluminum.	Fe^2+^ at (5 mg L^−1^)	2.8–2.9	Pilot scale	Neither plant growth nor the immobilization of *D. magna* were affected by the presence of CECs nor their transformation products after solar photo-Fenton. Therefore, toxic effects were associated to dissolved organic matter and its oxidation products. The post-treatment reduced toxicity and phytotoxicity through time.	(Michael et al., 2019)
*Daphnia magna* and *Aliivibrio fischeri* (acute toxicity), *Raphidocelis subcapitata* (chronic toxicity).	UP and MSE (CAS)	CTC	Irradiated volume of 10.24 L, consisted of 8 acrylic glass tubes (length of 150 cm, external diameter of 3.3 cm, and thickness of 0.25 cm).	Fe^2+^ (5.6 mg L^−1^) Fe^3+^-EDDS (5.6:58 mg L^−1^)	Natural	Pilot scale	Photo-Fenton performance was toxic to *A. fischeri* < *R. subcapitata* < *D. magna*. Toxicity to *R. subcapitata* decreased from 100 % up to 53 % (solar photo-Fenton) and from 57 % to 38 % (solar photo-Fenton with EDDS) within 300 min of treatment. High toxicity levels (100 %) were detected for *D. magna* test for both treatments. None of the treatments were able to reduce toxicity to 50 % of initial effect, and toxicity increased as treatment time increased from 0 to 300 min for all endpoints.	(Maniakova et al., 2020)
*Aliivibrio fischeri* and *Drosophila melanogaster* flies (acute toxicity)	UP and MSE (UASB + PT)	Solar reactor	Conventional glass recipient depth: 4.9 cm; diameter: 15.5 cm, irradiated surface: 189 cm^2^, total volume: 500 mL Artificial irradiation by two black light lamps (10 W each, 350–400 nm) placed in parallel (3.5 cm) 1.0 cm above the surface	Fe^3+^-EDDS Molar ratio of 1:1; 1:2; 1:3	2.7 and 6.0	Laboratory scale	**UP**: inhibition of the bioluminescence emitted by *A. fischeri* at 13.6 kJ m^−2^ increased for both artificial and solar irradiation indicating the generation of toxic byproducts. Toxic effect to *A. fischeri* was eliminated within 41.5 kJ m^−2^ of treatment. **MWW**: Photo-Fenton treatment reduced the toxicity to *D. melanogaster* flies.	(Gonçalves et al., 2020)
*Daphnia magna* and *Aliivibrio fischeri* (acute toxicity), *Tetrahymena thermophila* (chronic toxicity), and *Spirodela polyrhiza* and *Lactuca sativa* (phytotoxicity)	MSE (CAS)	RPR	PVC, 0.98 m length and 0.36 m width separated by a central wall. The paddlewheel set at 200 rpm, mixing time of ~1.52 min.	Fe^2+^ (3 × 20 mg L^−1^)	Natural	Pilot Scale	Inhibition of *D. magna* by CAS effluent was equivalent to 20 %. After 20 min of photo-Fenton treatment, inhibition dropped to 5 %. *A. fischeri* bioassays presented no sensitivity before or after photo-Fenton. For chronic toxicity, inhibition of *T. thermophila* exposed to CAS effluent was equivalent to 40.9 %. After 20 and 90 min of photo-Fenton, no toxic effect was observed. CAS effluent showed stimulation of *L. sativa*, while solar photo-Fenton inhibited seeds growth after 20 min of treatment and no effect was observed within 90 min.	(Freitas et al., 2017)
*Daphnia magna* and *Aliivibrio fischeri* (acute toxicity), and *Tetrahymena thermophila* (chronic toxicity)	MSE (CAS)	CPC and RPR	**CPC**: Two twin reactor of Pyrex tubes, length of 1.5 m and diameter of 5 cm, volume of 7 L and illuminated volume of 2.1 L, mixing time of 5 min. **RPR**: maximum volume of 18 L, 5 cm liquid depth, of 0.98 m length and 0.37 m width. 3 min mixing time.	Fe^2+^ (20 mg L^−1^)	7.0	Pilot Scale	CAS effluent was not toxicity to *D. magna* nor *A. fischeri*. Likewise, there was no increase in toxicity for *D. magna* after solar photo-Fenton for both reactors. Meanwhile, there was an increase in luminescence after 30 min of treatment. Bioassays with *T. thermophila* showed an inhibitory effect of 13.8 % for CAS effluent, and photo-Fenton in the RPR had higher reduction upon chronic toxicity compared to CPC.	(Esteban García et al., 2018)

Note: MSE = municipal secondary effluent; UP = ultrapure water; CAS = conventional activated sludge; UASB + PT = upflow anaerobic sludge blanket reactor followed by post-treatment; CPC = compound parabolic collector; RPR = raceway pond reactor.

In the study conducted by Freitas et al. (2017), acute toxicity to *D. magna* increased after solar photo-Fenton compared to MSE before treatment due to the generation of toxic by-products. A slight decrease (6.7 %) in the immobilization of *Daphnids* could only be observed after 300 min of treatment. In this sense, the bioassay with *D. magna* was considered as a sensitive as an indicator of wastewater acute toxicity. In the same study, two endpoints were validated (germination index and root growth) for phytotoxicity of samples upon *Sinapis alba*, *Lepidium sativum*, and *Sorghum saccharatum* as test organisms (Michael et al., 2012).

The degradation of ampicillin, clarithromycin, erythromycin, ofloxacin, sulfamethoxazole, tetracycline, and trimethoprim present in MES (CAS and MBBR) by solar photo-Fenton was investigated along with the inhibition of plants growth (phytotoxicity) and immobilization of *D. magna*. According to the authors, neither antibiotics nor their transformation products affected toxicity, thus suggesting that by-products from DOM degradation induced toxicity (Michael et al., 2019). These results corroborate with findings obtained by Michael et al. (2012) and call attention to the need for more studies related to the toxic effects promoted by DOM oxidation byproducts during solar photo-Fenton. This is even more relevant in the context of MSE from UASB and UASB + PT systems, as they contain higher concentrations of organic matter. Therefore, future studies must analyze acute and chronic toxicity of wastewater samples obtained during the application of solar photo-Fenton as post-treatment of UASB and UASB + PT.

Acute toxicity to *Drosophila melanogaster* was evaluated to analyze the performance of solar photo-Fenton to treat UASB + PT (coagulation, flocculation, and flotation) using Fe^3+^-EDDS to remove a mix of 4 pesticides, atrazine (1.13 mg L^−1^), ametrine (1.08 mg L^−1^), imidacloprid (mg L^−1^), and tebuthiuron (1.14 mg L^−1^). In the absence of pesticides, UASB + PT effluent presented no toxicity to this organism. Yet, the spiked matrix promoted fly mortality. Photo-Fenton performed under artificial and sunlight irradiation decreased toxicity. Although these results indicate the feasibility of performing solar photo-Fenton with effluents from UASB + PT, most UASB systems operating in LA are usually applied alone or followed by a simple and less costly post-treatment stage, such as trickling filter, rather than coagulation + flocculation + flotation. Although the referred study did not focus in pharmaceutical drugs, it was the only one which assessed post-treatment of MSE from UASB system. So, more studies evaluating the toxicity of UASB effluents before and after photo-Fenton, including other trophic levels, must still be investigated.

Maniakova et al. (2020) evaluated the efficiency of the solar photo-Fenton process, aiming at CECs removal from MSE along with acute and chronic toxicity responses. Despite satisfactory reduction of CECs, none of the treatments reduced the toxicity to *D. magna*. In addition, the applied treatment increased toxicity for all endpoints. Moreover, this work demonstrated that *D. magna* (acute toxicity, consumer) is more sensitive than *R. subcapitata* (chronic toxicity, producer), which is more sensitive than *A. fischeri* for this kind of sample. These results emphasize the need to evaluate toxicity for different trophic levels.

Although chronic toxicity assay using *D. magna* was sensitive to detect the effect of MSE treated via solar photo-Fenton, bioassays with *A. fischeri* did not reveal any effects (Freitas et al., 2017). This is probably because the latter is an acute toxicity test, while MSE contains a reduced concentration of chemical compounds and organic matter after solar photo-Fenton, thus being more likely to promote chronic toxic effects. On the other hand, solar photo-Fenton removed chronic toxicity to *T. thermophila* (Esteban García et al., 2018). Considering higher concentration of organic matter and lack of data regarding toxicity of UASB and UASB + PT effluents, acute and chronic toxicity tests should be performed for MSE characterization before the definition of the most appropriate bioassays to evaluate post-treatment performance.

In contrast to observations made by Michael et al. (2012), the work performed by Freitas et al. (2017) reproduced the methodology proposed by Young et al. (2012) to calculate phytotoxicity responses in *L. sativa* exposed to MSE before and after solar photo-Fenton. In the adapted method, the calculated relative growth index (RGI) expresses results as inhibition, stimulation, or non-significant effects upon root elongation, thus enabling comparisons among different operation conditions. Concerning solar photo-Fenton application as post-treatment of CAS effluent, only a stimulation of root elongation was detected within 90 min (Freitas et al., 2017). Although results obtained from this bioassay provide essential information considering the reuse of treated wastewater for irrigation in agriculture (Sobrero and Ronco, 2009), the classification of toxic effects concerning the RGI has only been applied in a few studies for environmental matrices such as: synthetic wastewater before and after UASB system (Rodrigues-Silva et al., 2022b), MSE (Freitas et al., 2017), pesticides contaminated water (Utzig et al., 2019), antibiotic contaminated water (Rodrigues-Silva et al., 2022a), and clinical laboratory hospital wastewater (Sá et al., 2021).

The photo-Fenton process performed for the treatment of UASB + PT effluent with different iron complexes using H_2_O_2_ or S_2_O8^2−^ as oxidants (Table 1; Silva et al., 2021a) was evaluated as according to antibacterial activity (AA) towards a model organism DH5α (*E. coli* strain) to assess the feasibility of applying treated wastewater for irrigation without significant impacts upon soil microbial biodiversity. Photo-Fenton performed with Fe^3+^-citrate and H_2_O_2_ was capable of eliminating AA within 7 kJ m^−2^, while S_2_O_8_^2−^ increased the accumulated energy required for AA elimination (31 kJ m^−2^). In this regard, Gonçalves et al. (2020) also studied the photo-Fenton process (Fe^3+^-EDDS and H_2_O_2_) as post-treatment of UASB + PT (Table 2), confirming the capacity of this advanced treatment to eliminate AA. Nevertheless, it is critical to conduct studies to evaluate solar photo-Fenton efficiency for post-treatment of UASB effluent and UASB followed by biological polishing systems.

Overall, despite the importance of evaluating toxicity during AOPs applied as post-treatment of MSE, this study reveals that there is a lack of data on this direction, especially when it comes to studies conducted with UASB effluent and at pilot-scale. Future studies should investigate the minimum treatment time and accumulated irradiation required to remove toxicity of real MSE to different trophic levels.

## 5 Disinfection by solar photo-Fenton as post-treatment of municipal wastewater treatment plant effluent at pilot-scale

Solar photo-Fenton has been proved as an effective process for the disinfection of MSE. Mechanisms acting on the disinfection during solar photo-Fenton are associated to a cascade of events promoted by radiation alone and oxidative radicals formed in the bulk which damage external and internal cell constituents. The baseline solar process (solar disinfection or SODIS) acts on the inactivation of crucial enzymes responsible for protecting cells against oxidative stress, thus leading to the accumulation of reactive oxygen species (ROS) and resulting in bacteria death. In summary, bacteria inactivation via SODIS may take place by the action of UV irradiation, intracellular oxidative damage, and thermal heating (Pichel et al., 2019). According to Giannakis (2018a), sunlight provides UV-A radiation (320–400 nm) which increases bacteria cell membrane permeability and induces the generation of ROS. Also, UV-B radiation (280–315 nm) comprising solar spectrum directly damages DNA (Feng et al., 2020). In addition to these mechanisms, DOM, NO^3+^, carbonate, and other ions present in MSE may lead to the formation of oxidative radicals after exposure to irradiation, thus promoting external cell damage (Serna-Galvis et al., 2019).

According to Dutta et al. (2019), AOP-driven disinfection includes the destruction of bacteria cell wall, cell membrane, enzymes, and intracellular genetic material. The primary components of bacteria cells (phospholipids and proteins) are protective barriers, which blocks the entrance of extracellular substances into cells. ROS produced in AOPs attack this cell envelope allowing for a free pass of ROS to the inner cell compartment (Wang et al., 2021). Once in the inner cell compartment, ROS oxidize fatty acids, leading to lipid-peroxyl radicals and forming a chain reaction (Xiao et al., 2019). Although autoxidation of enzymes (co-enzyme A, Ahp Alkyl hydroperoxide reductase, glutathione reductase, superoxide dismutase, catalase, and hydroperoxidases) protects against ROS in cells, oxidative species generated in AOPs systems lead to enzyme oxidation, thus inhibiting microbial cell respiration and resulting in bacterial death. Also, ROS can destroy genetic components (DNA and RNA) (Chen et al., 2021; Dutta et al., 2019).

The addition of H_2_O_2_ as an oxidant in the Fenton reaction may promote an imbalance of the internal ROS regulation system. In addition, iron present in the bulk plays an inactivation mechanism caused by the ligand-to-metal charge transfer (Giannakis et al., 2016) and can also form complexes with negatively charged microorganisms in Fenton-based processes at natural pH. For instance, *Escherichia coli*, presents positively and negatively charged constituents in its membrane which may react with iron (Giannakis, 2018b).

Considering higher concentration of solids in UASB effluent compared to CAS effluent and scattering light effect promoted by these constituents (Fig. 2C, D), it is likely that the effect of sunlight alone (SODIS) upon disinfection in these matrices will be reduced when compared to the effect observed in CAS effluents. After all, disinfection via photo-Fenton is an intracellular process initiated by effects promoted by sunlight. Yet, as this kick-off stage may be hindered in UASB effluents due to turbidity, transportation of these reagents into the bacterial cell may be limited, disabling bacteria inactivation by the photo-Fenton process (Giannakis et al., 2018). So, higher concentrations of Fe^2+^ and H_2_O_2_ may be required for the inactivation of bacteria to allow for disinfection of MSE from UASB (Uhl et al., 2015) when compared to CAS effluent. Table 4 summarizes main results obtained in studies that evaluated the disinfection promoted by solar photo-Fenton performed on CAS effluent.

**Table 4:** Disinfection of municipal wastewater treatment plant effluent from conventional activated sludge by solar photo-Fenton.

Target bacteria	Concentration	Reactor	Reactor features	Iron Source	pH	Experimental scale	Results	Reference
Total coliforms (TC), *E. coli*, and *Enterococcus* sp.	TC 3.5 10^6^ CFU mL^−1^. *E. coli* 2.8 10^4^ CFU mL^−1^	CPC	Two twin reactors made of Pyrex tubes, 1.5 m length, 5 cm diameter and 2.5 mm thickness. Fit onto two CPC mirrors, each 0.21 m^2^ illuminated surface.	Fe^2+^ (20 mg L^−1^ 50 mg L^−1^)	Natural	Pilot scale	Photo-Fenton inactivated TC and *E. coli* within 45 min. Regarding the evaluation of the HCO_3_^−^, TC was inactivated within 80 min for both natural and adjusted HCO_3_^−^, although *E. coli* was completely removed after 80 min for the natural HCO_3_^−^ and at 40 min when the HCO_3_^−^ concentration was reduced to 100 ± 5 mg L^−1^. Treatment performed at circumneutral pH was not effective to reduce natural bicarbonate. Sequential iron addition strategy (20–10–10 mg L^−1^) reduced the disinfection rate compared to the single iron addition. Treatment with 100 mg L^−1^ of H_2_O_2_ removed *E. coli* within 30 min and TC within 45 min.	(Ortega-Gómez et al., 2014)
Total coliforms (TC), *E. coli*	TC 7.3 10^4^ CFU mL^−1^, and *E. coli* 1.8 10^3^ CFU mL^−1^	RPR	PVC, 0.98 m length and 0.36 m width separated by a central wall. The paddlewheel set at 200 rpm, mixing time of ~1.52 min.	Fe^2+^ (3 × 20 mg L^−1^)	Natural	Pilot scale	The work was the first to report wastewater disinfection by solar photo-Fenton in RPR. Complete inactivation of enteric wild bacterial was achieved within 90 min.	(Freitas et al., 2017)
Total coliforms (TC), *E. coli* and *Enterococcus* sp.	TC 3.5 10^6^ CFU mL^−1^, *E. coli* 2.8 10^4^ CFU mL^−1^*Enterococcus* sp. 7.1 10^3^ CFU mL^−1^	CPC and RPR	**CPC**: Two twin reactors, 1.5 m length and 5 cm diameter, 7 L of total volume and 2.1 L of illuminated volume, 5 min mixing time. **RPR**: maximum volume of 18 L, 5 cm liquid depth, 0.98 m length and 0.37 m width. 3 min of mixing time.	Fe^2+^ (20 mg L^−1^)	7.0	Pilot scale	80 min of treatment reached TC < 1 CFU mL^−1^ for both reactors. *E. coli* and *Enterococcus* sp. in the CPC, reached <1 CFU mL^−1^ within 70 min, while 80 min were required in the RPR. This slight difference in terms treatment time required for effective disinfection may be due to higher irradiance registered for the CPC when compared to the RPR.	(Esteban García et al., 2018)
Total coliforms (TC), *E. coli* and *Enterococcus* sp.	TC 6.4 10^5^ CFU mL^−1^, *E. coli* 1.4 10^4^ CFU mL^−1^*Enterococcus* sp. 3.6 10^3^ CFU mL^−1^	RPR	PVC. 0.98 m length of and 0.37 m width of, and 5 cm liquid depth. Maximum working volume of 18 L, and mixing time of 3 min.	Fe^2+^ (20 mg L^−1^)	7.0	Pilot-Scale	At least 1 kJ L^−1^ was needed to inactivate bacteria to levels under the LOD. H_2_O_2_ residual concentration above 30 mg L^−1^ hindered regrowth. Yet, regrowth was evident for residual H_2_O_2_ below 15 mg L^−1^. A HRT of 30 min did not remove bacteria concentration below 1 CFU mL^−1^, reaching this goal at 60 min for *E. coli.* 60 min of HRT did.	(De la Obra Jiménez et al., 2019)
*E. coli* and Antibiotic Resistant Bacteria (ARB)	*E. coli* 4.1 10^6^ MPN 100 mL^−1^	RPR	1.22 m maximum length, 1.02 m central wall length and 0.20 m width; total volume of 12 L; 5 cm liquid depth	Fe^2+^ (5 × 11 mg L^−1^)	7.0	Pilot scale	***E. coli***: solar/Fe/S_2_O_8_^2−^ was more effective (3 log units; QUV = 1.9 kJ L^−1^) than solar/Fe/H_2_O_2_ (2.5 log units; QUV = 2.5 kJ L^−1^). **ARB**: removal of strains resistant to ampicillin, chloramphenicol, erythromycin, amoxicillin, sulfadiazine, sulfamethoxazole, and trimethoprim + sulfamethoxazole was higher via solar/Fe/S_2_O_8_^2−^ compared to solar/Fe/H_2_O_2_, reaching a maximum of 3 log units for bacteria resistant to ampicillin and amoxicillin.	(Starling et al., 2021a)

Note: CPC = compound parabolic collector; RPR = raceway pond reactor; HRT = hydraulic retention time.

Rommozzi et al. (2020) evaluated the effect of inorganic ions (HCO_3_^−^, Cl^−^, SO_4_^2−^, NO_2_^−^, NO_3_^−^, and NH_4_^+^) at concentrations equivalent to those which occur in natural waters upon the disinfection of *Escherichia coli*. The authors demonstrated that the generation of HO• by excitation of NO_2_^−^ and NO_3_^−^ (indirect solar photolysis) played a prominent role in *E. coli* inactivation via solar disinfection. However, for the solar photo-Fenton process performed with Fe^2+^ at neutral pH, the HO• generated through the oxidative treatment was transformed into other radical species in the presence of ions. In this sense, matrix complexity and wastewater composition must be considered prior to the application of AOPs not only considering the removal of CECs (Lado Ribeiro et al., 2019), yet also their effect upon disinfection (Rizzo et al., 2019). Considering high concentration of organic matter and ions in UASB and UASB + PT effluents (Fig. 2E, F), it is critical to evaluate the efficiency of solar photo-Fenton for disinfection of this type of effluent.

Carbonate and bicarbonate ions present in MSE act as scavengers of hydroxyl radicals during photo-Fenton treatment, generating carbonate radicals (Lado Ribeiro et al., 2019; Pignatello et al., 2006). Previous studies have shown that addition of concentrated acid to MSE prior to solar photo-Fenton treatment promotes a reduction in the concentration of these anions and avoids this undesired effect (Klamerth et al., 2009). Besides, bicarbonates may promote iron precipitation, thus leading to the use of increased iron concentrations (Carra et al., 2013). According to Esteban García et al. (2018), initial concentration of total inorganic carbon (TIC) must be under 50 mg L^−1^ to enable MSE treatment by photo-Fenton. Even so, inactivation of enteric bacteria via solar photo-Fenton in a CPC was similar in the presence of bicarbonates concentrations raging from to 100 mg L^−1^ to 250 mg L^−1^ (Table 3) (Ortega-Gómez et al., 2014). These results are favorable for the application of solar photo-Fenton for disinfection of UASB effluents which contain high concentrations of radical scavengers. Furthermore, Starling et al. (2021b) showed that the choice to work with alternative radicals such as SO_4_^−^• in a modified solar photo-Fenton system is an attractive option since this radical is more selective and less reactive towards wastewater natural scavengers compared to HO•.

The first research to report disinfection by solar photo-Fenton in a RPR demonstrated complete removal of wild enteric bacteria, total coliforms and *E. coli* within 90 min of treatment with no regrowth after treatment (Freitas et al., 2017). Solar photo-Fenton performed at natural pH showed complete inactivation of wild bacteria present in MSE after 80 min of treatment in RPR and CPC. As investment costs associated to the CPC are 40 times higher compared to the RPR, lower prices were obtained for disinfection of CAS effluent via solar photo-Fenton in a RPR (Esteban García et al., 2018). However, as MSE from UASB systems show higher color and turbidity, the CPC may be more advantageous for disinfection of UASB and UASB + PT effluents, as it allows for concentration of incident irradiation.

Continuous operation of solar photo-Fenton in a RPR at pilot-scale and neutral pH was evaluated as according to MSE disinfection (De la Obra Jiménez et al., 2019). The authors presented that at least 1 kJ L^−1^ of accumulated UVA energy is required to inactivate total coliforms, *E. coli*, and *Enterococcus* sp. to levels below 1 CFU mL^−1^. In this perspective, a HRT of 30 min was the most efficient to promote disinfection, producing 305 m^3^ m^−2^ year^−1^ of treated effluent (Almeria, Spain weather conditions). Moreover, a kick-off treatment in batch operation mode (20 mg L^−1^ of Fe^2+^ and 50 mg L^−1^ of H_2_O_2_; 120 min) followed by continuous operation mode (20 mg L^−1^ of Fe^2+^ and 30 mg L^−1^ of H_2_O_2_) avoided excess of H_2_O_2_ in the steady-state. Nonetheless, to the best of our knowledge, no studies have evaluated the application of solar photo-Fenton for disinfection of MSE effluents from other types of biological processes rather than conventional activated sludge.

Although reduced HRT is sufficient for CEC removal from MSE via solar photo-Fenton (Table 1), toxicity and disinfection must also be investigated during treatment to determine the most suitable HRT to allow for safe discharge or reuse of wastewater. All of these variables were asses in the study conducted by Starling et al. (2021a) (Table 3) who evaluated simultaneous degradation of CECs and toxicity response (*A. fischeri*) along with *E. coli* disinfection and removal of antibiotic-resistant bacteria (ARB). Solar photo-Fenton at pilot scale in a RPR using H_2_O_2_ as an oxidant removed 2.5 log units of *E. coli*, while persulfate mediated solar photo-Fenton removed 3.0 log units. Moreover, both processes (solar photo-Fenton and persulfate mediated solar photo-Fenton) decreased bacteria strains resistant to ampicillin, chloramphenicol, erythromycin, amoxicillin, sulfadiazine, sulfamethoxazole, and trimethoprim + sulfamethoxazole.

## 6 Concluding remarks and future research

As UASB systems stand out for their vast application aiming at municipal sewage treatment in LA, this review aimed to analyze challenges associated to post-treatment of UASB system effluents via solar photo-Fenton in light of (i) physicochemical characterization of MSE originated from aerobic and anaerobic systems and (ii) results obtained in previous research associated to the application of solar photo-Fenton as post-treatment of MSE. A statistical comparison of physicochemical characterization data reported for MSE originated from CAS, UASB and UASB + TF detected significant differences between these effluents. This comparative analysis showed that UASB effluents are more complex than CAS effluent as they contain higher concentrations of organic and inorganic matter, total suspended solids, turbidity and alkalinity. Considering that NOM and carbonate ions are known as hydroxyl radical scavengers which compete with target CECs, these features may impose challenges to the use of solar photo-Fenton as post treatment of UASB effluents.

Nevertheless, literature on the application of solar photo-Fenton as post-treatment of MSE from UASB systems is scarce. As these treatments are highly applied in developing countries, a few strategies that may overcome challenges imposed by inherent features of UASB effluents were highlighted in this review, such as: using higher accumulated energy in the irradiated treatment, adding pre-treatment stages to remove suspended solids and avoid sunlight scattering, and promoting carbonate and bicarbonate stripping before the application of the oxidative process. For instance, the influence of inorganic matter present in UASB effluent to the effectiveness of solar photo-Fenton at circumneutral pH should be studied in detail as it was done for inorganic constituents of the CAS effluent (Lado Ribeiro et al., 2019). This will allow for the establishment of strategies to overcome limitations imposed by UASB affluent alkalinity.

Although recent studies have explored the use of chelating agents, mainly Fe^3+^-EDDS, to allow for the application of solar photo-Fenton as post-treatment of MSE at neutral pH, this strategy has not yet been studied for post-treatment of UASB effluents. However, considering costs and unknown toxicity associated to chelating agents, the introduction of Fe^2+^ in fractionated additions during treatment is seen as a more feasible strategy for solar photo-Fenton application in emerging countries. Still, future studies must compare treatment performance and costs associated to solar photo-Fenton conducted at circumneutral pH using Fe^3+^-EDDS versus fractionated additions of Fe^2+^. Also, coming research should assess the application of natural organic matter present in waste from industrial processes as complexing agents in Fenton-based processes applied as post-treatment of UASB effluents.

Regarding solar photoreactors, although the CPC and RPR have been extensively investigated for the conduction of solar photo-Fenton as post-treatment of MSE form aerobic treatment systems in pilot scale, and even in real scale, no studies have reported the use of these solar photoreactors for the treatment of MSE from UASB systems. Such studies are required to conduct a cost-benefit analysis considering investment, implementation, and operation costs which are critical for decision making in tropical developing countries that lack investments in sanitation infrastructure.

Considering that toxic transformation products may be formed during MSE treatment via solar photo-Fenton, this review also puts together outcomes reported in different studies which analyze the effects of MSE treated by solar photo-Fenton by in vivo assays and in silico tools. It is important to highlight that those toxic byproducts may be formed either from the oxidation of CECs or natural organic matter inherent to MSE. Considering differences in the organic content occurring in CAS and UASB effluents and lack of studies on solar photo-Fenton as post-treatment of UASB effluents, it is critical to conduct in vivo and in silico approaches to assess the toxicity of UASB effluents during solar photo-Fenton treatment. In addition, these studies must be performed for different test organisms as different trophic levels interact differently with matrix components.

Finally, another aspect raised in this review is associated to the efficiency of solar photo-Fenton on the disinfection of MSE. This is critical as there is a tendency for increased reuse of treated wastewater in irrigation worldwide and solar photo-Fenton may be an alternative to reach reuse standards. However, only a few studies have investigated the removal of CECs, toxicity and disinfection from CAS effluent simultaneously during solar photo-Fenton while none have assessed all these endpoints for UASB effluents. There is also a gap in the literature concerning the removal of ARB and ARG in post-treatment of UASB systems effluents. These represent opportunities to be tackled by future research leading to advances in the application of solar photo-Fenton as post-treatment of MSE in LA.

## CRediT authorship contribution statement

**Fernando Rodrigues-Silva**: Conceptualization, Methodology, Formal Analysis. Investigation, Writing – Original Draft & Review and Editing, Visualization. **Maria Clara V.M. Starling**: Conceptualization, Methodology, Writing – Original Draft & Review and Editing, Visualization, Supervision, Project Administration, Funding Acquisition. **Camila C. Amorim**: Conceptualization, Methodology, Resources, Writing – Original Draft & Review and Editing, Visualization, Supervision, Project Administration, Funding Acquisition.

## Declaration of competing interest

The authors declare that they have no known competing financial interests or personal relationships that could have appeared to influence the work reported in this paper.

## Data Availability

No data was used for the research described in the article.
